# Birth Weight and Stroke in Adult Life: Genetic Correlation and Causal Inference With Genome-Wide Association Data Sets

**DOI:** 10.3389/fnins.2020.00479

**Published:** 2020-06-11

**Authors:** Ting Wang, Zaixiang Tang, Xinghao Yu, Yixing Gao, Fengjun Guan, Chengzong Li, Shuiping Huang, Junnian Zheng, Ping Zeng

**Affiliations:** ^1^Department of Epidemiology and Biostatistics, School of Public Health, Xuzhou Medical University, Xuzhou, China; ^2^Department of Biostatistics, School of Public Health, Medical College of Soochow University, Suzhou, China; ^3^Department of Pediatrics, Affiliated Hospital of Xuzhou Medical University, Xuzhou, China; ^4^Center of Stroke and Department of Cardiology, Affiliated Hospital of Xuzhou Medical University, Xuzhou, China; ^5^Cancer Institute, Xuzhou Medical University, Xuzhou, China; ^6^Center of Clinical Oncology, Affiliated Hospital of Xuzhou Medical University, Xuzhou, China; ^7^Jiangsu Center for the Collaboration and Innovation of Cancer Biotherapy, Cancer Institute, Xuzhou Medical University, Xuzhou, China

**Keywords:** birth weight, stroke and subtypes, ischemic stroke, Mendelian randomization, maternal effect, causal association, genetic correlation, fetal origins

## Abstract

**Objective:**

Prior studies have shown that there is an inverse association between birth weight and stroke in adulthood; however, whether such association is causal remains yet known and those studies cannot distinguish between the direct fetal effect and the indirect maternal effect. The aim of the study is to untangle such relationship using novel statistical genetic approaches.

**Methods:**

We first utilized linkage disequilibrium score regression (LDSC) and Genetic analysis incorporating Pleiotropy and Annotation (GPA) to estimate the overall genetic correlation between birth weight and stroke. Then, with a set of valid birth-weight instruments which had adjusted fetal and maternal effects, we performed a two-sample Mendelian randomization (MR) to evaluate its causal effect on stroke based summary statistics from large scale genome-wide association study (GWAS) (*n* = 264,498 for birth weight and 446,696 for stroke). We further validated the MR results with extensive sensitivity analyses.

**Results:**

Both LDSC and GPA demonstrated significant evidence of shared maternal genetic foundation between birth weight and stroke, with the genetic correlation estimated to −0.176. However, no fetal genetic correlation between birth weight and stroke was detected. Furthermore, the inverse variance weighted MR demonstrated the maternally causal effect of birth weight on stroke was 1.12 (95% confidence interval [CI] 1.00–1.27). The maternal ORs of birth weight on three subtypes of stroke including cardioembolic stroke (CES), large artery stroke (LAS) and small vessel stroke (SVS) were 1.16 (95% CI 0.93–1.43), 1.50 (95% CI 1.14–1.96) and 1.47 (95% CI 1.15–1.87), respectively. In contrast, no fetal causal associations were found between birth weight and stroke or the subtypes. Those results were robust against extensive sensitivity analyses, with Egger regression ruling out the possibility of pleiotropy and multivariable MR excluding the likelihood of confounding or mediation effects of other risk factors of stroke.

**Conclusion:**

This study provides empirically supportive evidence on the fetal developmental origins of stroke and its subtypes. However, further investigation is warranted to understand the pathophysiological role of low birth weight in developing stroke.

## Introduction

Stroke is primarily caused by brain infarction (i.e., ischemic stroke) or intracerebral hemorrhage (ICH) with a neurological deficit of sudden onset (Malik et al., 2018), and it represents one of the leading causes of morbidity and mortality worldwide (Kyu et al., 2018; Roth et al., 2018). Although conventional risk factors (e.g., smoking) for stroke have been well-established; the pathological mechanism of stroke remains yet completely understood. Recently, the role of early fetal growth even before birth was recognized and the hypothesized adverse determinants *in utero* are related to the risk of developing stroke in adulthood (Eriksson et al., 2000) – a hypothesis referred to as the fetal origins of adult chronic diseases which was first proposed in the late 1980s (Barker, 1990; Lucas et al., 1999; Barker et al., 2002). This hypothesis for fetal developmental programming states the fetus has to adapt to adverse intrauterine exposures (e.g., under-nutrition) by slowing the growth of body, consequently, resulting in low birth weight (a commonly employed index of exposure on early growth for intrauterine environment). However, such adaptation has a long-term influence on postnatal health status because developmental programming *in utero* can permanently alter organ structure (e.g., liver, heart, and kidney) and modify epigenetic regulation of gene expression. The brain can be directly modulated due to the sparing adaptation in restricted fetal growth (Eriksson et al., 2000). Such recognition has revolutionized the understanding of pathogenesis for many adult chronic metabolic diseases. Many observational studies have provided evidence showing low birth weight associated with enhanced susceptibility to stroke in later life (Rich-Edwards et al., 1997; see Supplementary Table S1 for more information).

However, it remains unclear whether the observed association between birth weight and stroke in prior studies uncover a truly causal association, or only a spurious correlation due to confounding emerging during the prenatal/postnatal life (Barker, 1990; Leon, 1998; Eriksson et al., 2000; Law, 2002; Ruiz-Narvaez et al., 2014; Kahn et al., 2017) or due to pleiotropy and shared genetic foundation (Rich-Edwards et al., 1997; Lawlor et al., 2005). More importantly, the observed inverse association between birth weight and stroke can be driven by the indirect maternal effect and/or direct fetal effect (Horikoshi et al., 2013, 2016; Beaumont et al., 2018; Warrington et al., 2019). To our knowledge, none of previous studies had distinguished the maternal effect from the fetal effect. The ability to discriminate relatively maternal and fetal genetic contributions to birth weight (Warrington et al., 2018, 2019) makes it feasible to deeper investigate the origin of the inverse relationship between birth weight and stroke. In addition, it is also unknown whether such negative association can be generalized to stroke subtypes because stroke is a complex heterogeneous disease with multiple subtypes having distinct differences in clinical manifestation and genetic background (Malik et al., 2018).

In the past few years, several large scale genome-wide association studies (GWASs) have been performed and have greatly advanced our understanding of the genetic architecture for birth weight (Horikoshi et al., 2013, 2016; Beaumont et al., 2018; Warrington et al., 2019) and stroke (Malik et al., 2018). It has been shown that birth weight shares specific single nucleotide polymorphisms (SNPs) with many adult diseases (Horikoshi et al., 2016; Warrington et al., 2019). However, little is known about the shared heritability and overall pleiotropy between birth weight and stroke, as well as its subtypes. Quantifying the extent to which the two types of phenotypes share genetic origin will shed some light on common biological mechanism underlying birth weight and stroke and provides novel insights into the relationship between them.

Herein, our main objectives are to investigate the genetic overlap and to further determine the causal relationship between birth weight and stroke. To achieve those objectives, we performed a comprehensive genetic analysis with linkage disequilibrium score regression (LDSC) (Bulik-Sullivan et al., 2015) and Mendelian randomization (MR) (Sheehan et al., 2008) based on summary statistics obtained from large scale GWASs [*n* = ∼300,000 for birth weight (Warrington et al., 2019) and ∼447,000 for stroke (Malik et al., 2018)].

## Materials and Methods

### GWAS Genetic Data Sources for Birth Weight and Stroke

We initially obtained summary association statistics for birth weight from the Early Growth Genetics (EGG) Consortium^1^ (Warrington et al., 2019). The association analysis was performed on each SNP for own (*n* = 298,142) and offspring (*n* = 210,267) birth weights after controlling covariates (e.g., gestational age). To distinguish the fetal and maternal genetic components of genotypes on birth weight, an efficient structural equation model (SEM) was implemented up to 264,498 individuals who reported their own birth weight and 179,360 individuals who reported their offspring birth weight (Warrington et al., 2018), generating adjusted fetal effects after controlling for the maternal genotype and maternal effects after controlling for the offspring’s genotype. It was shown some SNPs had only a direct fetal effect, some had only an indirect maternal effect and some had a combined effect of the two on birth weight (Warrington et al., 2019; Supplementary Figure S1). The separation of fetal and maternal effects of SNPs on birth weight plays a crucial role in clarifying the origin of the observed relationship between birth weight and stroke. In our LDSC analysis we applied the genome-wide fetal and maternal specific effects of birth weight (see below for details); in our MR analysis we employed a set of independent associated SNPs (*p* < 5.00E-8) reported in Warrington et al. (2019); Supplementary Tables S2, S3.

We next yielded summary association statistics for any ischaemic stroke (AIS) at the MEGASTROKE Consortium^2^ (Malik et al., 2018). In addition, we also obtained summary statistics for three stroke subtypes: cardioembolic stroke (CES), large artery atherosclerotic stroke (LAS) and small vessel stroke (SVS). The genetic data sets employed in our analysis are briefly summarized in Table 1.

**TABLE 1:** GWAS genetic data sets used in the present study.

Traits	*n* (or case/control)	Data source
Birth weight (fetal or maternal)	264,498 (own) and 179,360 (offspring)	Warrington et al., 2019
AIS	40,585/406,111	Malik et al., 2018
CES	9,006/406,111	Malik et al., 2018
LAS	6,688/406,111	Malik et al., 2018
SVS	11,710/406,111	Malik et al., 2018

*AIS: any ischaemic stroke; CES: cardioembolic stroke; LAS: large artery atherosclerotic stroke; SVS: small vessel stroke.*

### Estimation of Overall Genetic Correlation Between Birth Weight and Stroke With LDSC and GPA

To assess shared polygenic architecture between birth weight and stroke, we applied the cross-trait LDSC to calculate the genetic correlation (Bulik-Sullivan et al., 2015). LDSC quantifies the genome-wide genetic overlap with summary statistics only while taking into account LD structure among genetic variants, and can be easily conducted by regressing the product of the *z* statistics of two traits against the LD scores that are computed from the 1000 Genomes project phase III (The 1000 Genomes Project Consortium, 2015). The regression slope provides an unbiased estimate for genetic correlation between phenotypes (Bulik-Sullivan et al., 2015). Detail of quality control procedure for birth weight and stroke in the LDSC analysis is shown in Supplementary Material.

To complement LDSC we also conducted the recently proposed GPA analysis (**G**enetic analysis incorporating **P**leiotropy and **A**nnotation) (Chung et al., 2014), which can provide additional results about pleiotropy between the two phenotypes. Let *π*_10_ denotes the probability that a SNP is associated with the first phenotype but not the second, *π*_01_ denotes the probability that a SNP is associated with the second phenotype but not the first, *π*_11_ denotes the probability that a SNP is associated with both phenotypes and *π*_00_ denotes the probability that a SNP is not associated with any phenotypes. Then GPA aims to estimate these proportions that characterize the SNP causal effects to better understand the relationship between the phenotypes. In particular, two important ratio quantities, *π*_11_/(*π*_10_ + *π*_11_) or *π*_11_/(*π*_01_+*π*_11_) are estimated, representing the proportion of SNPs associated with one phenotype that are also associated with the other and indicating the extent of common biological pathways to which the two phenotypes may share (Chung et al., 2014; Zeng et al., 2018). As complex correlations among SNPs can bias the estimate of GPA, we employed a LD-based pruning method to remove large correlations between pairs of SNPs [using PLINK (Purcell et al., 2007) based on the European individuals in the 1000 Genomes project phase III (The 1000 Genomes Project Consortium, 2015): indep-pairwise 100 25 0.2] to ensure the remaining SNPs were not in high LD with each other in the GPA analysis.

### Estimation of Causal Effects Between Birth Weight and Stroke With Two-Sample MR Analysis

**FIGURE 1:**
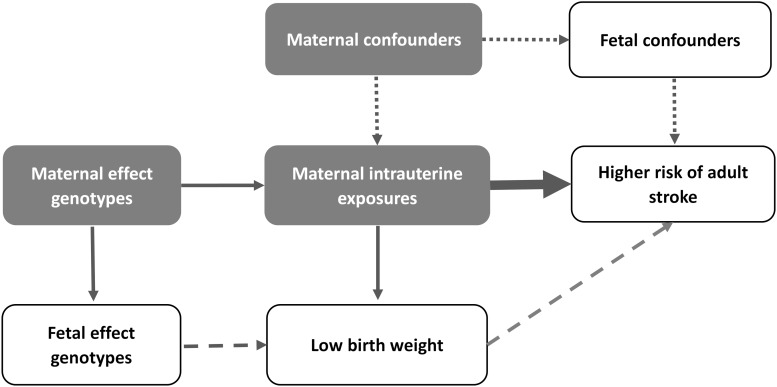
Overview of our idea in the present study. The thin arrows represent the relationship between SNPs and maternal intrauterine exposures; the thick arrow represents the causal effect of interest; the dotted arrows represent the potential confounding that are not associated with the genetic instrument; the dashed arrows represent the fetal effect.

We first aligned to birth weight-increasing allele with that of stroke. Then the causal effect of birth weight on stroke was estimated with inverse-variance weighted (IVW) MR methods (Burgess et al., 2017a; Hartwig et al., 2017) based on fetal or maternal specific effects of SNPs (Figure 1; Warrington et al., 2019). We then calculated the odds ratio (OR) for every one unit decrease of birth weight, with the unit estimated to be 488 g across all the sub-studies in a recent GWAS of birth weight (Horikoshi et al., 2016). We also generated informative plots (e.g., SNP effects scatter) for further illustrating our MR results.

To assess whether MR model assumptions were violated in our analysis and to assess the robustness of the results, we performed several complementary and sensitivity analyses for the causal effect estimation of birth weight on stroke: (1) weighted median-based method to estimate the causal effect when some of the instrument variables of birth weight are likely invalid (Bowden et al., 2016a); (2) leave-one-out (LOO) cross-validation analysis (Noyce et al., 2017) and MR-PRESSO analysis (Verbanck et al., 2018) for pleiotropy and outlier instrument detection; (3) MR-Egger regression to evaluate the directional pleiotropy of instruments (Bowden et al., 2016b; Burgess and Thompson, 2017); (4) IVW estimation for causal effects of birth weight on the three stroke subtypes; (5) multivariable MR analysis (Do et al., 2013; Burgess and Thompson, 2015; Burgess et al., 2017b) to investigate whether other relevant complex phenotypes (i.e., 12 early growth traits, 7 anthropometric traits, 14 metabolic traits and 9 socioeconomic traits) may mediate or confound the causal effect of birth weight on stroke. (6) Statistical power for MR analysis was calculated with an analytic approach proposed by Brion et al. (2013)^3^. Details of power calculation and the multivariable MR analysis are shown in Supplementary Material. The MR analyses were performed within the R (version 3.5.2) software and the significance level was set to 0.05.

## Results

### Estimated Overall Genetic Correlation Between Birth Weight and Stroke

Linkage disequilibrium score regression shows there is a negative maternal genetic correlation between birth weight and stroke (*r*_*g*_ = −0.176, *p* = 1.10E-3), in contrast to the positive but non-significant fetal genetic correlation (*r*_*g*_ = 0.007, *p* = 0.886). However, due to the small sample size that leads to negative estimates of heritability for the stroke subtypes, we cannot acquire a valid estimate for maternal or fetal genetic correlations between birth weight and the subtypes of stroke (Supplementary Table S4).

Using GPA and based on ∼137,000 approximately independent SNPs, we observe substantially maternal pleiotropy exists between birth weight with stroke and its subtypes (except SVS; Table 2); however, no fetal pleiotropy is detected (except SVS; Table 2), in line with the results of LDSC shown above. In addition, we find the proportion of SNPs that are associated with both birth weight and stroke (i.e., *π*_11_) and the proportion of SNPs that are associated with birth weight are also associated with stroke (i.e., *π*_11_/(*π*_10_ + *π*_11_) or *π*_11_/(*π*_01_ + *π*_11_)) are consistently larger for the maternal specific effect of birth weight compared with those for the fetal specific effect of birth weight (Table 2).

**TABLE 2:** Pleiotropy estimated among the fetal/maternal specific effect of birth weight and stroke as well as its subtypes.

Stroke	*π*_00_	*π*_10_	*π*_01_	*π*_11_	*π*_11_/(*π*_10_ + *π*_11_)	*π*_11_/(*π*_01_ + *π*_11_)	LRT	*p*-value
**Fetal effect of birth weight**								
AIS	0.789	0.128	0.058	0.024	0.158	0.293	3.32	0.068
CES	0.748	0.169	0.071	0.012	0.066	0.145	0.16	0.689
LAS	0.721	0.196	0.057	0.026	0.117	0.313	0.66	0.418
SVS	0.790	0.127	0.045	0.038	0.230	0.458	5.95	0.015
**Maternal effect of birth weight**								
AIS	0.807	0.105	0.042	0.047	0.309	0.528	22.62	1.97E−06
CES	0.772	0.139	0.051	0.037	0.210	0.420	5.87	0.015
LAS	0.731	0.180	0.047	0.041	0.186	0.466	4.59	0.032
SVS	0.759	0.152	0.074	0.015	0.090	0.169	1.50E−03	0.969

*The last two columns are the likelihood ratio test (LRT) statistic and *p*-value of hypothesis testing for pleiotropy between birth weight and stroke. π_10_, the proportion of SNPs that are associated with stroke; π_01_, the proportion of SNPs that are associated with birth weight; π_11_/(π_10_ + π_11_), the proportion of SNPs that are associated with stroke are also associated with birth weight; π_11_/(π_01_ + π_11_), the proportion of SNPs that are associated with birth weight are also associated with stroke.*

### Estimated Causal Effects for Birth Weight on Stroke

**FIGURE 2:**
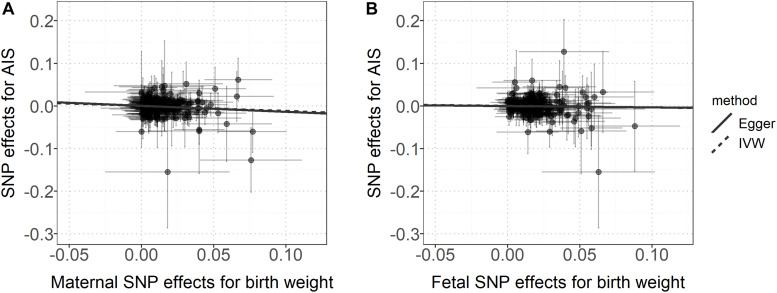
**(A)** Relationship between the maternal effect of birth weight and the effect size estimates on stroke for all instruments. **(B)** Relationship between the fetal effect of birth weight and the effect size estimates on stroke for all instrument. The 95% confidence intervals for the estimated SNP effect sizes on stroke are shown as vertical lines, while the 95% confidence intervals for the estimated SNP effect sizes on birth weight are shown as horizontal lines.

**FIGURE 3:**
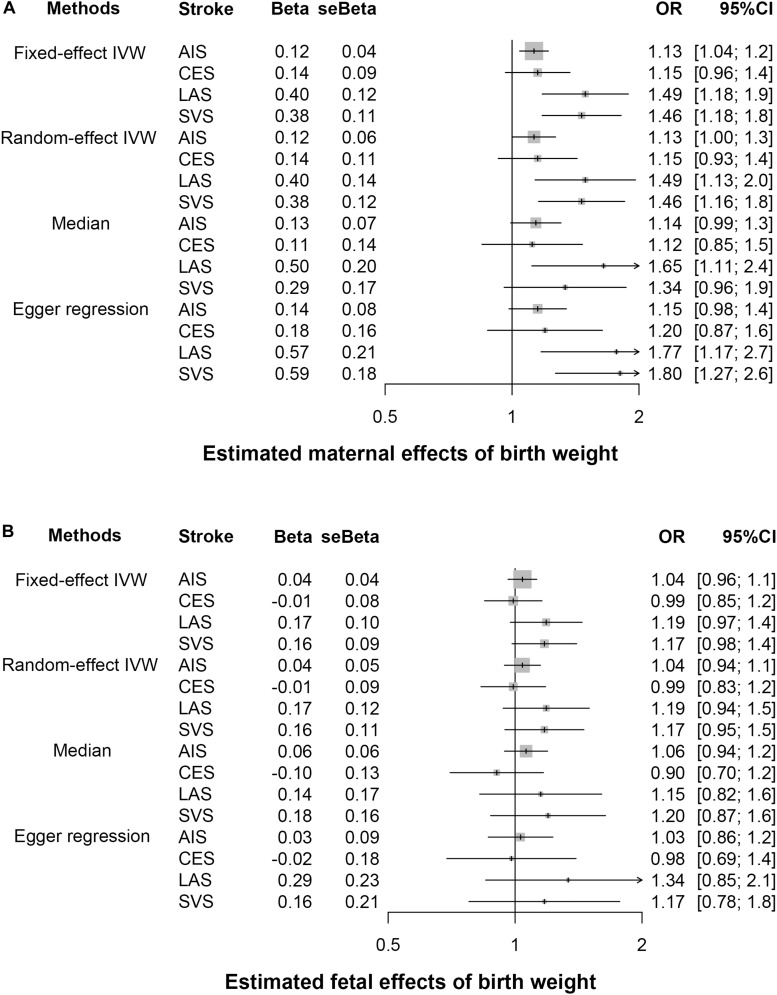
Estimated causal effects of birth weight (**A**, maternal effect; **B**, fetal effect) on stroke and its subtypes. Four MR methods (the fixed- and random-effects IVW method, weighted median method and Egger regression) were performed. AIS, any stroke; LAS, large artery atherosclerotic stroke; CES, cardioembolic stroke; SVS, small vessel stroke.

Owing to the presence of heterogeneity across instrumental variables (Cochran’s Q *p* < 0.05 for all the IVW MR analyses), the random-effects IVW method was thus utilized. We find the maternal causal effect of birth weight on stroke is 1.13 (*p* = 0.040), indicating a 13% (95% CI 0–27%) increase of stroke risk for every 488 g decrease in birth weight (Figures 2A, 3). The maternal ORs of birth weight on CES, LAS and SVS are 1.15 (95% CI 0.93–1.43, *p* = 0.182), 1.49 (95% CI 1.13–1.96, *p* = 3.49E-3), and 1.46 (95% CI 1.16–1.85, *p* = 1.90E-3), respectively, again implying lower birth weight is associated with an increased risk of stroke subtypes. However, no statistically significant fetal causal associations are identified between birth weight and stroke or the three subtypes (Figures 2B, 3).

### Sensitivity Analyses for MR

We next performed multiple sensitivity analyses to evaluate the robustness of this inversely maternal association between birth weight and stroke above (Figure 3 and Supplementary Figures S2, S3). The weighted median approach generates a similar maternal causal effect to the random-effects IVW method (OR = 1.14, 95% CI 0.99–1.31, *p* = 0.077). The LOO analysis shows no single instrument substantially influences the estimated maternal casual effect. For example, after removing rs45446698 (located within gene *CYP3A7-CYP3AP1* and had the largest maternal effect size on birth weight with beta = 0.077), the OR is estimated to be 1.11 (95% CI 0.99–1.24, *p* = 0.068); after removing rs139429176 (located within gene *P2RX7/HNF1A* and had the largest effect size on stroke with beta = −0.155), the OR is estimated to be 1.12 (95% CI 1.00–1.25, *p* = 0.041). Like the LOO analysis, MR-PRESSO also demonstrates no maternal instrumental outliers at the significance level of 0.05. The intercept of the MR-Egger regression is 5.08E-4 (se = 1.35E-3, *p* = 0.707), indicating horizontal pleiotropy unlikely biases the estimated maternal causal effect. Nevertheless, we screened the EBI website^4^ and removed 50 instruments that were associated with other relevant traits and diseases (Supplementary Table S5), and still obtained similar estimates (OR = 1.11, 95% CI 1.00–1.22, *p* = 0.039 for AIS; OR = 1.07, 95% CI 0.87–1.32, *p* = 0.513 for CES; OR = 1.53, 95% CI 1.17–1.99, *p* = 0.002 for LAS and OR = 1.42, 95% CI 1.11–1.82, *p* = 0.006 for SVS).

**FIGURE 4:**
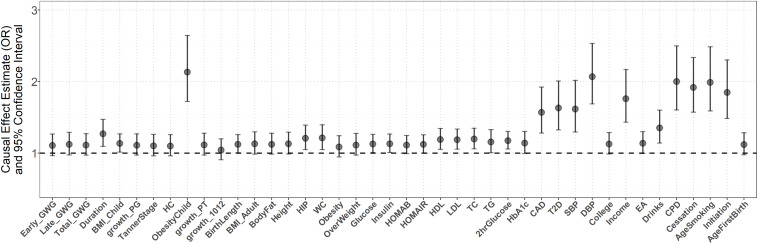
Estimated maternal causal effects of birth weight on stroke using the multivariate MR regression.

The multivariable MR analysis rules out the possibility that early growth and adult complex traits can completely mediate or confound the maternal causal effect (Figure 4). Note that, adjustment for some of traits (e.g., growth 1012) attenuates the maternal causal effect of birth weight on stroke, while adjustment for some of traits (e.g., obesity in children, SBP, or DBP) strengthens such causal effect and adjustment for some of traits (e.g., overweight and fasting glucose) results in little difference. In particular, we find the control of gestational duration cannot essentially weaken the estimated maternal causal effect (the adjusted OR = 1.27, 95% CI 1.09–1.47, *p* = 1.88E-3) although the duration of gestation is a major determinant of birth weight and is highly correlated with birth weight (Lawlor et al., 2005), implying the maternal causal effect of birth weight is independent of gestational duration. Additional sensitivity analyses demonstrate the estimated maternal or fetal causal effects for CES, LAS, and SVS are also robust against alternative MR methods, instrumental outliers and pleiotropy (Figure 3 and Supplementary Figures S2, S3).

### Power Calculation for Our MR Analysis

Finally, we calculated the statistical power for our MR analysis and supposed the true fetal or maternal causal OR of lower birth weight on stroke was 1.20 (approximately equal to the estimated effect in the present study), the proportion of variance explained by instrumental variables was equal to 2% (approximately equal to the heritability explained by associated SNPs reported in Warrington et al., 2019), the significance level *α* is 0.05 and the proportion of stroke (or subtype) cases is the same as that given in Table 1. Using this method, the power of detecting such causal effect was calculated to be 100% for AIS, 75% for CES, 63% for LAS, and 85% for SVS, respectively, implying we have an adequately high capacity for identifying the causal association and also suggesting the detected maternal causal effects of birth weight on stroke and its subtypes are truly positive and the non-significant fetal causal effects of birth weight on stroke and its subtypes are unlikely falsely negative.

## Discussion

In the present study, we have investigated genetic correlation and causal association between birth weight and stroke. Because birth weight is subject to both fetal and maternal genomes with diverse effects in orientation and magnitude, as a particular case, it is essential to distinguish fetal and/or maternal components of instrumental effects on birth weight in genetic analysis; otherwise the results would be biased or even misleading. However, due to unavailability of relevant data sources, prior studies cannot distinguish such important components. By taking full advantage of the latest GWAS genetic datasets having adjusted fetal and maternal effects of birth weight for each genetic variant, the present analysis holds the capability that better untangles the source of the negative relationship between birth weight and stroke.

### Summary of Our Study

Our analysis provides new supportive evidence on the maternal molecular genetic overlap between birth weight and stroke, and further offers robust evidence showing lower birth weight, by maternal rather than fetal genome, is causally associated with stroke and its subtypes in later life. Furthermore, such effect is independently of many other possible risk factors of stroke. In contrast, our analysis does not support the fetal genetic correlation and the direct fetal causal effect of birth weight on stroke or its subtypes, further demonstrating the separate maternal influence. *A priori*, our study is in favor of the fetal developmental origins of stroke as well as its subtypes and offers the scientific evidence for intervening adverse intrauterine environments (e.g., appropriate nutritional additions for pregnant women). In view of the increasing survival rate of fetus with low birth weight today, our result therefore has important implications on the early predictor of stroke in adulthood. In addition, the present analysis also further confers the significance to unmask the fetal and maternal contributions respectively to birth weight as an exposure (or outcome) in genetic studies.

### Comparison of Our Findings With Those in Previous Studies

We now compare our results with those displayed in previous work (Supplementary Table S1). Our findings are complementary to and consistent with previous studies where inverse genetic associations were identified between birth weight and other adult diseases (e.g., *r*_*g*_ = −0.27 for T2D and −0.30 for T2D) (Horikoshi et al., 2016). The causality results are also consistent with findings reported in many prior studies, including a longitudinal American cohort of women nurses (Rich-Edwards et al., 1997), a retrospective Finland study on men (Eriksson et al., 2000), a historical Swedish cohort in both men and women (Hyppönen et al., 2001) as well as other relevant studies (Lawlor et al., 2005; Rich-Edwards et al., 2005). However, compared with those studies, our study has the following advantages. (1) Prior results in those observational studies might be easily biased by potential confounders (e.g., maternal cigarette smoking and alcohol drinking during the prenatal period, or gestational diabetes mellitus) (Lawlor et al., 2005). In contrast, our findings are less susceptible to those confounding factors as MR depends on the principle that the random meiotic assortment of genotypes is independent of confounders and disease process of stroke in adulthood. (2) Compared with prior studied which had small sample size (e.g., generally less than 1,000), our study includes much larger cases of stroke (up to ∼41,000); therefore, sufficient statistical power is guaranteed. (3) This study relied on summary association statistics and simultaneously investigated both stroke and its subtypes, holding wider implications on the relationship between birth weight and stroke. (4) Previous studies demonstrated association between birth weight and stroke/subtypes, but cannot establish causality. In contrast, MR can be thought of as a naturally randomized controlled trial (Hingorani and Humphries, 2005) and thus provides empirical evidence for the causal relationship between birth weight and stroke. (5) Importantly and as demonstrated before, unlike prior work which cannot distinguish between maternal and fetal influences on stroke, we have the ability of revealing birth weight has an indirect maternal, but not a direct fetal, causal effect on stroke.

### Other Contributions of the Present Study

Our study, at least in part, provides answers for several previous unsolved questions. First, our analysis showed the shared maternal genetic foundation between birth weight and stroke cannot fully explain the observed inverse association, partly resolving the question proposed in Rich-Edwards et al. (1997). However, further work is warranted to identify the maternal or fetal specific genetic factors that influence both birth weight and stroke in later life. Second, even after removing the indirect influence of early growth traits (or other relevant socioeconomic traits and adult lifestyle) on stroke, birth weight still has a direct role on the development of stroke in adulthood, suggesting there exist other unknown pathways from which birth weight is associated with stroke (Rich-Edwards et al., 1997). Third, the present MR study further distinguishes adverse intrauterine environments, rather than the direct fetal effect, has an indirect long-term influence on the risk of stroke and its subtypes. Forth, as birth weight in our study was within in the normal range (the 95% limit is 2,492–4,405 g in terms of Horikoshi et al., 2016), the identified inverse maternal causal effect on stroke is not likely driven by individuals born at the extremes of birth weight.

### Limitations of Our Study

First, due to the small sample sizes for the subtypes of stroke and relying on summary association results, we cannot determine the fetal or maternal genetic correlation between birth weight and stroke subtypes. Investigations with much larger sample size for stroke subtypes are warranted. Second, like most other MR applications, we assumed a linear relationship between birth weight and stroke. It is certainly possible that there may be a non-linear relationship. However, as most of the individuals had their birth weights within in the normal range, the linearity assumption in our MR is likely reasonable (Rich-Edwards et al., 1997). Third, although no *a priori* hypothesis concerning sex differences in the effect of birth weight on stroke exists, it was shown that there may be gender specific causal effects of birth weight on stroke with the effect of lower birth weight on stroke slightly higher in females compared with in males (Lawlor et al., 2005). However, due to the dependence on of GWAS summary association statistics, we cannot further conduct stratified analysis to estimate the causal effects of birth weight on stroke by gender. Because of the same reason of unavailability of genetic datasets, we also cannot explore the relationship between extremely very low/high birth weight and stroke. Fifth, our study focuses only on European population; it is not known whether our findings can be generalized to other populations. Sixth, we limited our study on ischemic stroke; it is unclear whether our discovers can be applicable to hemorrhagic stroke.

## Data Availability Statement

Publicly available datasets were analyzed in this study. This data can be found here: https://egg-consortium.org/; "https://strokegenetics.org/.

## Author contributions

PZ, ZT, SH, and JZ conceived the idea for the study. PZ and TW obtained the genetic data. PZ, XY, YG, and TW performed the data analyses. PZ, TW, XY, FG, CL, and SH interpreted the results of the data analyses. All the authors wrote the manuscript.

## Conflict of Interest

The authors declare that the research was conducted in the absence of any commercial or financial relationships that could be construed as a potential conflict of interest.

## Footnotes

1 https://egg-consortium.org/

2 https://strokegenetics.org/

3 https://cnsgenomics.shinyapps.io/mRnd/

4 https://www.ebi.ac.uk/
