# Supplementary material for: Birth Weight and Stroke in Adult Life: Genetic Correlation and Causal Inference With Genome-Wide Association Data Sets
Source: Front Neurosci. 2020 Jun 11;14:479. doi: 10.3389/fnins.2020.00479 (PMC7301963; doi:10.3389/fnins.2020.00479)
Supplement: Supplementary file 1 [file Data_Sheet_1.docx]

**Supplementary Texts and Results**

Inverse variance weighted method

Methodologically, the inverse variance weighted (IVW) Mendelian randomization is equivalent to weighted linear regression ([DerSimonian and Laird, 1986](#_ENREF_13);[Thompson and Sharp, 1999](#_ENREF_38);[Brockwell and Gordon, 2001](#_ENREF_5);[Burgess et al., 2017](#_ENREF_7);[Burgess and Thompson, 2017](#_ENREF_9);[Hartwig et al., 2017](#_ENREF_17);[Yavorska and Burgess, 2017](#_ENREF_44)). To see this, let the estimate of effect size for instrumental variable *i* be $\hat{\beta}_{i}^{BW}$ (*i* = 1, 2, …, *k*) for the exposure birth weight (BW), with *k* the number of instrumental variables; let the outcome be stroke and the effect size estimate and variance for the same instrumental variable *i* on stroke be $\hat{\beta}_{i}^{Stroke}$ and$\text{Var}(\hat{\beta}_{i}^{Stroke})$. Then, we have

$$\hat{\beta}_{i}^{Sroke} = \hat{\beta}_{i}^{BW}c + \varepsilon_{i}, \varepsilon_{i} \sim N(0, \text{λVar}(\hat{\beta}_{i}^{Stroke}))$$

where *c* is the causal effect of birth weight on stroke and λ is the residual error variance. According to , with the weighted least squares method, we can obtain the estimate of *c*

$$\hat{c} = \frac{\sum_{i=1}^{k} \text{Var}(\hat{\beta}_{i}^{Stroke})^{-1}\hat{\beta}_{i}^{Stroke}\hat{\beta}_{i}^{BW}}{\sum_{i=1}^{k} \text{Var}(\hat{\beta}_{i}^{Stroke})^{-1}(\hat{\beta}_{i}^{BW})^{2}}$$

with variance

$$\text{Var}(\hat{c}) = \frac{\hat{\lambda}}{\sum_{i=1}^{k} \text{Var}(\hat{\beta}_{i}^{Stroke})^{-1}(\hat{\beta}_{i}^{BW})^{2}}$$

where $\hat{\text{λ}}$ is the estimated residual variance from . Note that, when $\hat{\text{λ}}\text{=}1$ the estimation is referred to as fixed-effects IVW; when $\hat{\text{λ}}\text{>}1$ the estimation is referred to as random-effects IVW.

Multivariable Mendelian randomization analysis

We also perform the multivariable Mendelian randomization analysis ([Burgess and Thompson, 2015](#_ENREF_8);[Zeng and Zhou, 2019a](#_ENREF_46);[b](#_ENREF_47)) to evaluate the independent effect after adjusting some covariates. For example, both birth weight and systolic blood pressure (SBP) were assumed to have effects on stroke; we try to estimate the relation between birth weight and stroke while controlling for SBP

$$\hat{\beta}_{i}^{Stroke} = \hat{\beta}_{i}^{BW}c^{BW}+\hat{\beta}_{i}^{SBP}c^{SBP}+ \varepsilon_{i}, \varepsilon_{i} \sim N(0, \text{λVar}(\hat{\beta}_{i}^{Stoke}))$$

where $\hat{\beta}_{i}^{Stroke}$, $\hat{\beta}_{i}^{BW}$ and $\hat{\beta}_{i}^{SBP}$ are the marginal effects of SNP on Stroke, BW, and SBP, and $\varepsilon_{i}$ represents the residual error with variance λ. Again, using the weighted least squares method we can yield the estimate of $c^{BW}$.

Power calculation

We utilized an online calculator ([Brion et al., 2013](#_ENREF_4)) to calculate the test power with the input of several parameters (i.e. proportion of cases, odds ratio of the outcome variable per standard deviation of the exposure variable, sample size and proportion of variance in exposure variable explained by SNPs). The non-centrality parameter is calculated by

$$NCP=nR^{2}\frac{\left[P\left(\frac{OR}{1+P(OR-1)}-1\right)\right]^{2}}{P\left(1-P\right)-\left[P\left(\frac{OR}{1+P(OR-1)}-1\right)\right]^{2}}$$

where *P* is proportion of cases; *N* is total sample size, *OR* is True odds ratio of the outcome variable per standard deviation of the exposure variable; *R^2^* is proportion of variance in exposure variable explained by SNPs. Then the power is calculated as

$$1 - \beta= 1 - P(\text{χ}_{df, NCP}^{2'} \text{>} \text{χ}_{df, 1-\alpha}^{2})$$

where *β* is the type-II error; $\text{χ}_{df,NCP}^{2'}$ is a random variable from a non-central χ^2^ distribution with *df* degrees of freedom (*df* = 1) and $\text{χ}_{df, 1-\alpha}^{2}$ is the threshold of a central χ^2^ distribution for type-I error rate of *α*.

Quality control procedures for LDSC

Following prior studies ([Bulik-Sullivan et al., 2015](#_ENREF_6)), we performed stringent data quality control procedures for LDSC: (1) excluded non-biallelic SNPs and SNPs with strand-ambiguous alleles; (2) excluded SNPs that have no *rs* labels as well as duplicated ones; (3) excluded all SNPs that are located within the genetic region of major histocompatibility complex (chr6: 28,500,000-33,500,000); (4) kept only SNPs that were included within the 1000 Genomes project phase III while removing SNPs whose alleles did not match those in the 1000 Genomes project phase III ([The 1000 Genomes Project Consortium, 2015](#_ENREF_37)). Using a 10 Mb window, we computed the LD scores for 7,120,251 common SNPs (with the minor allele frequency larger than 0.01 and the p value of the Hardy Weinberg equilibrium test greater than 1E-5) on 503 European individuals in the 1000 Genomes project phase III ([The 1000 Genomes Project Consortium, 2015](#_ENREF_37)).

Traits used in multivariable analysis

In the multivariable analysis, we considered **twelve** early growth traits (i.e. early, late or total gestational weight gain (GWG) of offspring ([Warrington et al., 2018](#_ENREF_40)), gestational duration ([Liu et al., 2019b](#_ENREF_23)), birth length ([van der Valk et al., 2015](#_ENREF_39)), childhood obesity ([Bradfield et al., 2019](#_ENREF_3)), childhood BMI ([Felix et al., 2016](#_ENREF_16)), growth 1012 (a single height measurement at age 10 in girls and 12 in boys which targets the take-off phase of the growth spurt), growth PG (the total amount of growth across the pubertal growth period) and growth PT (the total amount of growth in late adolescence, targeting the timing of peak height growth velocity) ([Cousminer et al., 2013](#_ENREF_11)), head circumference (HC) ([Taal et al., 2012](#_ENREF_36)) and tanner stage ([Cousminer et al., 2014](#_ENREF_12))), **seven** anthropometric traits (i.e. adult BMI ([Yengo et al., 2018](#_ENREF_45)), height ([Yang et al., 2012](#_ENREF_43)), body fat percentage ([Lu et al., 2016](#_ENREF_24)), adult obesity and overweight ([Berndt et al., 2013](#_ENREF_2)), waist and hip circumference (WC and HIP) ([Shungin et al., 2015](#_ENREF_34))), **fourteen** metabolic traits (i.e. fasting glucose, fasting insulin and indices of *β*-cell function (HOMA-B) and insulin resistance (HOMA-IR) ([Dupuis et al., 2010](#_ENREF_14)), high density lipoprotein (HDL), low density lipoprotein (LDL), total cholesterol (TC) and triglycerides (TG) ([Willer et al., 2013](#_ENREF_42)), glucose level 2 hours after an oral glucose challenge (2hr-glucose) ([Saxena et al., 2010](#_ENREF_32)), glycated hemoglobin (HbA1c) ([Wheeler et al., 2017](#_ENREF_41)), coronary artery disease (CAD) ([Nikpay et al., 2015](#_ENREF_25)) and type 2 diabetes (T2D) ([Scott et al., 2017](#_ENREF_33)), and diastolic blood pressure (DBP) and systolic blood pressure (SDP) from the UK BioBank ([Bycroft et al., 2018](#_ENREF_10))) and **nine** socioeconomic traits (i.e. college ([Rietveld et al., 2013](#_ENREF_30)), educational attainment (EA) ([Rietveld et al., 2014](#_ENREF_29)), maternal age of first birth ([Barban et al., 2016](#_ENREF_1)), household income ([Hill et al., 2016](#_ENREF_18)), drinks per week, cigarettes per day, smoking cessation, age of smoking initiation and initiation of smoking ([Liu et al., 2019a](#_ENREF_22))). For all the complex traits, we obtained their association summary statistic for each SNPs in terms of the effect allele, marginal effect size estimate or log OR and standard error from public portals.


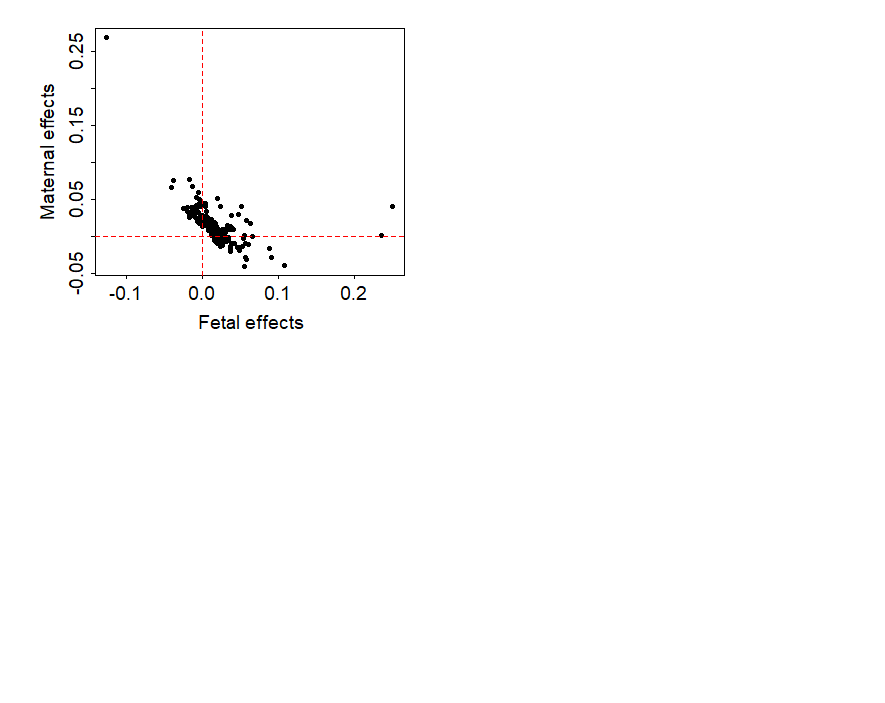


Figure S1. Relationship between the SEM-adjusted fetal effects and the SEM-adjusted maternal effects for the identified associated SNPs of birth weight (*p* < 5.0E-8). SEM: structural equation model.


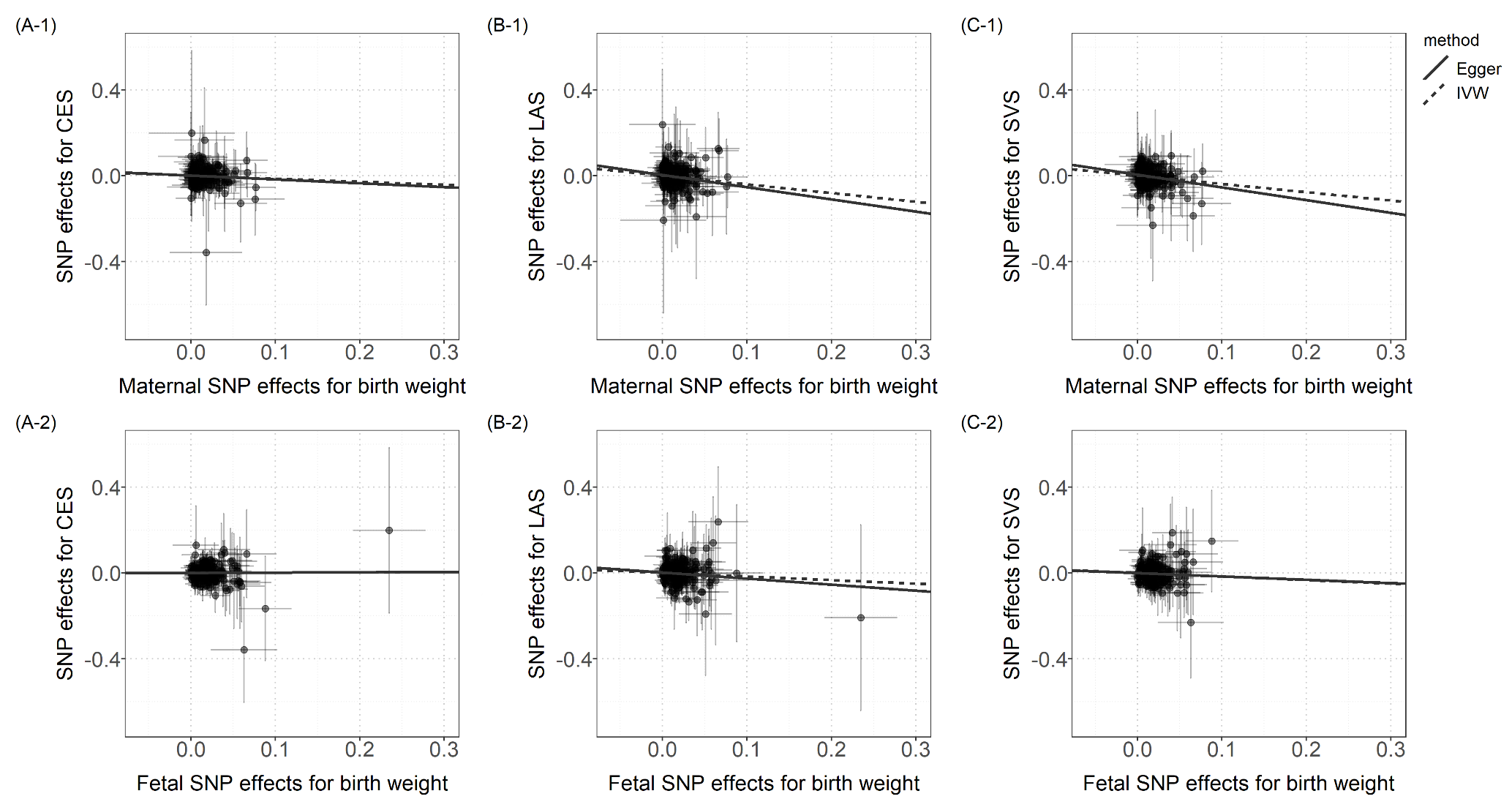


Figure S2. (A1-2) Relationship between the effect of birth weight and the effect size estimates on CES for all the instruments of birth weight. (B1-2) Relationship between the effect of birth weight and the effect size estimates on LAS for all the instruments of birth weight. (C1-2) Relationship between the effect of birth weight and the effect size estimates on SVS for all the instruments of birth weight. The top panel is for the fetal effect of birth weight and the bottom panel is for the maternal effect of birth weight. The 95% confidence intervals for the estimated SNP effect sizes on the subtypes of ischaemic stroke are shown as vertical black lines, while the 95% confidence intervals for the estimated SNP effect sizes on birth weight are shown as horizontal black lines. Note that, the instrumental outlier shown in the right side of plot A and B (i.e. rs138715366 on gene *YKT6/GCK*) does not influence the estimated fetal causal effect of birth weight on CES (OR = 1.00, 95% CI 0.83 - 1.19, *p* = 0.976) and LAS (OR = 1.17, 95% CI 0.93 -1.48, *p* = 0.179).


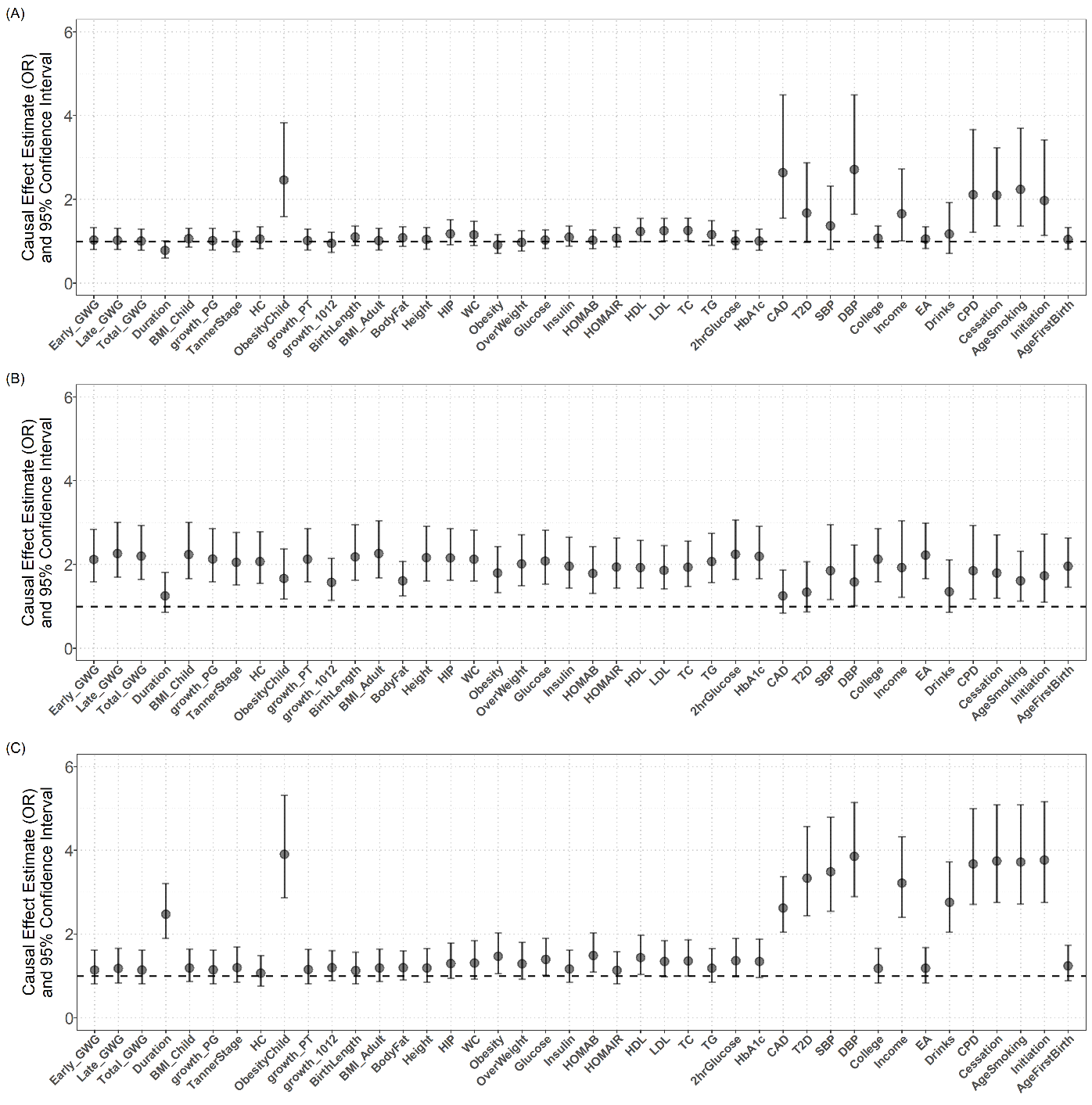


Figure S3. Estimated maternal causal effects of birth weight on CES (A), LAS (B) and SVS (C) using the multivariable MR regression. LAS: large artery atherosclerotic stroke; CES: cardioembolic stroke; SVS: small vessel stroke.

Table S1. Summary results for the association between birth weight and stroke extracted from selective previous studies

| Year | OR/HR | 95% CI | | *n* | Population | Birth weight variables | Ref |
| --- | --- | --- | --- | --- | --- | --- | --- |
|  |  | Lower | Upper |  |  |  |  |
| 1997 | 1.49 | 1.05 | 2.10 | 70,297 | American F | < 2268 g vs. 3175-3856 g | ([Richedwards et al., 1997](#_ENREF_28)) |
| 1997 | 1.25 | 0.98 | 1.61 | 70,297 | American F | 2268-2495 g vs. 3175-3856 g | ([Richedwards et al., 1997](#_ENREF_28)) |
| 1997 | 1.12 | 0.98 | 1.27 | 70,297 | American F | 2495-3175 g vs. 3175-3856 g | ([Richedwards et al., 1997](#_ENREF_28)) |
| 1997 | 0.96 | 0.80 | 1.15 | 70,297 | American F | 3856-4536 g vs. 3175-3856 g | ([Richedwards et al., 1997](#_ENREF_28)) |
| 1997 | 0.68 | 0.46 | 1.00 | 70,297 | American F | > 4536 g vs. 3175-3856 g | ([Richedwards et al., 1997](#_ENREF_28)) |
| 2000 | 2.60 | / | / | 3,639 | Finland M (Hemorrhagic stroke) | ≤ 2500 g vs. > 4000 g | ([Eriksson et al., 2000](#_ENREF_15)) |
| 2000 | 1.20 | / | / | 3,639 | Finland M (Hemorrhagic stroke) | 2501-3000 g vs. > 4000 g | ([Eriksson et al., 2000](#_ENREF_15)) |
| 2000 | 0.80 | / | / | 3,639 | Finland M (Hemorrhagic stroke) | 3001-3500 g vs. > 4000 g | ([Eriksson et al., 2000](#_ENREF_15)) |
| 2000 | 1.10 | / | / | 3,639 | Finland M (Hemorrhagic stroke) | 3501-4000 g vs. > 4000 g | ([Eriksson et al., 2000](#_ENREF_15)) |
| 2000 | 1.30 | / | / | 3,639 | Finland M (Thrombotic Stroke) | ≤ 2500 g vs. > 4000 g | ([Eriksson et al., 2000](#_ENREF_15)) |
| 2000 | 1.20 | / | / | 3,639 | Finland M (Thrombotic Stroke) | 2501-3000 g vs. > 4000 g | ([Eriksson et al., 2000](#_ENREF_15)) |
| 2000 | 1.10 | / | / | 3,639 | Finland M (Thrombotic Stroke) | 3001-3500 g vs. > 4000 g | ([Eriksson et al., 2000](#_ENREF_15)) |
| 2000 | 0.90 | / | / | 3,639 | Finland M (Thrombotic Stroke) | 3501-4000 g vs. > 4000 g | ([Eriksson et al., 2000](#_ENREF_15)) |
| 2001 | 0.61 | 0.45 | 0.83 | 10,853 | Sweden M&F (Hemorrhagic stroke) |  | ([Hyppönen et al., 2001](#_ENREF_19)) |
| 2001 | 0.80 | 0.77 | 1.03 | 10,853 | Sweden M&F (Thrombotic Stroke) |  | ([Hyppönen et al., 2001](#_ENREF_19)) |
| 2005 | 0.38 | 0.24 | 0.60 | 10,803 | Scotland | per 1-kg increase in birth weight | ([Lawlor et al., 2005](#_ENREF_21)) |
| 2005 | 1.23 | 0.76 | 1.98 | 66,111 | American Female | < 2268 g vs. 3175-3856 g | ([Rich-Edwards et al., 2005](#_ENREF_27)) |
| 2005 | 1.05 | 0.74 | 1.48 | 66,111 | American Female | 2268-2495 g vs. 3175-3856 g | ([Rich-Edwards et al., 2005](#_ENREF_27)) |
| 2005 | 1.16 | 0.98 | 1.36 | 66,111 | American Female | 2495-3175 g vs. 3175-3856 g | ([Rich-Edwards et al., 2005](#_ENREF_27)) |
| 2005 | 0.77 | 0.60 | 0.99 | 66,111 | American Female | 3856-4536 g vs. 3175-3856 g | ([Rich-Edwards et al., 2005](#_ENREF_27)) |
| 2005 | 1.28 | 0.89 | 1.85 | 66,111 | American Female | > 4536 g vs. 3175-3856 g | ([Rich-Edwards et al., 2005](#_ENREF_27)) |
| 2008 | 1.61 | 0.98 | 2.64 | 6,425 | Sweden M&F | < 1500 g vs. 3000-3499 g | ([Kaijser et al., 2008](#_ENREF_20)) |
| 2008 | 0.97 | 0.72 | 1.31 | 6,425 | Sweden M&F | 1500-1999 g vs. 3000-3499 g | ([Kaijser et al., 2008](#_ENREF_20)) |
| 2008 | 1.12 | 0.88 | 1.44 | 6,425 | Sweden M&F | 2000-2499 g vs. 3000-3499 g | ([Kaijser et al., 2008](#_ENREF_20)) |
| 2008 | 1.18 | 0.91 | 1.52 | 6,425 | Sweden M&F | 2500-2999 g vs. 3000-3499 g | ([Kaijser et al., 2008](#_ENREF_20)) |
| 2008 | 1.04 | 0.81 | 1.33 | 6,425 | Sweden M&F | 3500-3999 g vs. 3000-3499 g | ([Kaijser et al., 2008](#_ENREF_20)) |
| 2008 | 0.86 | 0.61 | 1.20 | 6,425 | Sweden M&F | ≥ 4000 g vs. 3000-3499 g | ([Kaijser et al., 2008](#_ENREF_20)) |
| 2009 | 0.83 | 0.64 | 1.08 | 31,307 | [Norway](http://www.baidu.com/link?url=tQ3J0MZA3m7J0yYiDnRnp5Sho-1xmBnAnn1PXppM3ZSieOLqATvUlM4uFC6j37sryrgwy4qwu94yCQbbUPNFKfKyGp-z3RNzWaGNtN4Vvk7) M&F | per 470 g increase in birth weight | ([Risnes et al., 2009](#_ENREF_31)) |
| 2009 | 0.80 | 0.59 | 1.08 | 31,307 | [Norway](http://www.baidu.com/link?url=tQ3J0MZA3m7J0yYiDnRnp5Sho-1xmBnAnn1PXppM3ZSieOLqATvUlM4uFC6j37sryrgwy4qwu94yCQbbUPNFKfKyGp-z3RNzWaGNtN4Vvk7) M | per 470 g increase in birth weight | ([Risnes et al., 2009](#_ENREF_31)) |
| 2009 | 0.90 | 0.56 | 1.42 | 31,307 | [Norway](http://www.baidu.com/link?url=tQ3J0MZA3m7J0yYiDnRnp5Sho-1xmBnAnn1PXppM3ZSieOLqATvUlM4uFC6j37sryrgwy4qwu94yCQbbUPNFKfKyGp-z3RNzWaGNtN4Vvk7) F | per 470 g increase in birth weight | ([Risnes et al., 2009](#_ENREF_31)) |
| 2011 | 0.57 | 0.37 | 0.88 | 23,689 | dizygotic twins | per 1-kg increase in birth weight | ([Öberg et al., 2011](#_ENREF_26)) |
| 2011 | 0.92 | 0.48 | 1.80 | 23,689 | monozygotic twins | per 1-kg increase in birth weight | ([Öberg et al., 2011](#_ENREF_26)) |
| 2016 | 1.37 | 1.06 | 1.76 | 63,815 | Hard ASCVD | <6 lbs vs 6 – 7 lbs 15 oz | ([Smith et al., 2016](#_ENREF_35)) |
| 2016 | 1.19 | 0.96 | 1.47 | 63,815 | Hard ASCVD | 8 – 9 lbs 15 oz vs 6 – 7 lbs 15 oz | ([Smith et al., 2016](#_ENREF_35)) |
| 2016 | 1.41 | 0.92 | 2.17 | 63,815 | Hard ASCVD | ≥ 10 lbs vs 6 – 7 lbs 15 oz | ([Smith et al., 2016](#_ENREF_35)) |
| 2016 | 1.33 | 1.18 | 1.51 | 63,815 | Any CVD | <6 lbs vs 6 – 7 lbs 15 oz | ([Smith et al., 2016](#_ENREF_35)) |
| 2016 | 1.03 | 0.92 | 1.15 | 63,815 | Any CVD | 8 – 9 lbs 15 oz vs 6 – 7 lbs 15 oz | ([Smith et al., 2016](#_ENREF_35)) |
| 2016 | 1.19 | 0.95 | 1.49 | 63,815 | Any CVD | ≥ 10 lbs vs 6 – 7 lbs 15 oz | ([Smith et al., 2016](#_ENREF_35)) |
| 2016 | 1.32 | 1.15 | 1.50 | 63,815 | Major CVD | <6 lbs vs 6 – 7 lbs 15 oz | ([Smith et al., 2016](#_ENREF_35)) |
| 2016 | 1.01 | 0.90 | 1.14 | 63,815 | Major CVD | 8 – 9 lbs 15 oz vs 6 – 7 lbs 15 oz | ([Smith et al., 2016](#_ENREF_35)) |
| 2016 | 1.22 | 0.97 | 1.56 | 63,815 | Major CVD | ≥ 10 lbs vs 6 – 7 lbs 15 oz | ([Smith et al., 2016](#_ENREF_35)) |

Table S2. Summary information of the SEM-adjusted fetal effects for the instrumental variables of birth weight.

| CHR | SNP | Position (hg19) | Gene | Effect Allele | Other Allele | Beta | Se |
| --- | --- | --- | --- | --- | --- | --- | --- |
| 1 | rs17367504 | 11,862,778 | *MTHFR* | A | G | 0.005 | 0.006 |
| 1 | rs2807319 | 22,554,176 | *WNT4* | G | A | 0.025 | 0.006 |
| 1 | rs4908404 | 28,691,734 | *PHACTR4* | T | C | 0.005 | 0.004 |
| 1 | rs12401656 | 43,456,767 | *FLJ32224/SLC2A1* | G | A | 0.029 | 0.006 |
| 1 | rs1937436 | 66,441,329 | *PDE4B* | G | A | 0.013 | 0.005 |
| 1 | rs2755253 | 67,470,843 | *SLC35D1/WDR78* | C | T | 0.010 | 0.004 |
| 1 | rs787541 | 68,051,230 | *GADD45A* | G | C | 0.007 | 0.004 |
| 1 | rs7525870 | 78,269,207 | *FAM73A* | G | A | 0.015 | 0.005 |
| 1 | rs41276588 | 118,148,384 | *FAM46C* | G | A | 0.002 | 0.005 |
| 1 | rs80278614 | 119,412,317 | *TBX15* | A | G | 0.052 | 0.009 |
| 1 | rs116276359 | 151,821,430 | *THEM5* | A | C | 0.049 | 0.012 |
| 1 | rs905938 | 154,991,389 | *DCST2/KCNN3* | C | T | 0.023 | 0.005 |
| 1 | rs670523 | 155,878,732 | *RIT1/LMNA* | G | A | 0.016 | 0.004 |
| 1 | rs144126567 | 161,510,516 | *FCGR3A* | C | G | 0.030 | 0.008 |
| 1 | rs72480273 | 161,644,871 | *FCGR2B/FCGR2C/HSPA6* | C | A | 0.022 | 0.005 |
| 1 | rs10913200 | 176,521,655 | *PAPPA2* | G | A | 0.038 | 0.013 |
| 1 | rs61830764 | 212,289,976 | *DTL* | A | G | 0.018 | 0.004 |
| 1 | rs7541039 | 214,176,779 | *PROX1* | T | C | 0.022 | 0.005 |
| 1 | rs3806315 | 214,724,668 | *PTPN14* | A | G | 0.016 | 0.004 |
| 1 | rs4655413 | 215,828,499 | *USH2A* | T | C | 0.012 | 0.005 |
| 1 | rs708122 | 228,216,997 | *WNT3A* | C | A | 0.015 | 0.004 |
| 2 | rs10495563 | 9,662,210 | *ADAM17* | A | G | 0.016 | 0.004 |
| 2 | rs2551347 | 23,912,401 | *KLHL29* | T | C | 0.029 | 0.005 |
| 2 | rs1179494 | 36,809,496 | *FEZ2* | G | C | 0.002 | 0.004 |
| 2 | rs754868 | 43,185,532 | *HAAO* | G | A | 0.019 | 0.004 |
| 2 | rs4952673 | 43,423,870 | *ZFP36L2* | G | A | 0.004 | 0.004 |
| 2 | rs17034876 | 46,484,310 | *EPAS1* | T | C | 0.039 | 0.005 |
| 2 | rs7596521 | 46,918,665 | *SOCS5* | A | G | 0.001 | 0.005 |
| 2 | rs186606513 | 97,482,001 | *CNNM3* | G | A | 0.047 | 0.016 |
| 2 | rs12104672 | 109,151,173 | *LIMS1* | T | G | 0.017 | 0.004 |
| 2 | rs11680809 | 113,570,809 | *IL1B* | A | C | 0.019 | 0.004 |
| 2 | rs12623454 | 121,326,297 | *LOC84931* | G | C | 0.015 | 0.004 |
| 2 | rs56188432 | 158,406,865 | *ACVR1C* | G | A | 0.250 | 0.049 |
| 2 | rs10173538 | 160,569,276 | *7-Mar* | C | T | 0.011 | 0.004 |
| 2 | rs560887 | 169,763,148 | *G6PC2* | T | C | 0.025 | 0.004 |
| 2 | rs139557015 | 182,072,326 | *MIR4437* | C | T | 0.041 | 0.012 |
| 2 | rs2280235 | 191,843,830 | *STAT1* | G | A | 0.014 | 0.005 |
| 2 | rs10181515 | 227,019,461 | *LOC646736/COL4A4/IRS1* | T | C | 0.021 | 0.005 |
| 3 | rs2574727 | 11,636,508 | *VGLL4* | A | G | 0.008 | 0.009 |
| 3 | rs9855896 | 14,287,150 | *LSM3* | A | G | 0.014 | 0.005 |
| 3 | rs60573957 | 14,884,409 | *FGD5* | C | T | 0.004 | 0.004 |
| 3 | rs2168443 | 46,947,087 | *PTH1R* | T | A | 0.010 | 0.004 |
| 3 | rs3965156 | 66,484,956 | *LRIG1* | A | C | 0.017 | 0.004 |
| 3 | rs73226528 | 113,398,642 | *KIAA2018* | T | C | 0.004 | 0.005 |
| 3 | rs11708067 | 123,065,778 | *ADCY5* | G | A | 0.056 | 0.005 |
| 3 | rs17315501 | 139,029,676 | *MRPS22* | A | G | 0.039 | 0.016 |
| 3 | rs6440006 | 141,142,691 | *ZBTB38* | G | A | 0.001 | 0.004 |
| 3 | rs2306700 | 142,123,841 | *XRN1* | T | C | 0.022 | 0.006 |
| 3 | rs10935733 | 148,622,968 | *CPA3/AGTR1* | T | C | 0.021 | 0.004 |
| 3 | rs4679760 | 155,855,418 | *KCNAB1* | C | G | 0.009 | 0.004 |
| 3 | rs1482852 | 156,798,294 | *LOC339894/CCNL1* | A | G | 0.054 | 0.004 |
| 3 | rs11711420 | 183,349,010 | *KLHL24* | T | G | 0.022 | 0.005 |
| 3 | rs11545169 | 184,020,542 | *PSMD2* | G | T | 0.020 | 0.006 |
| 4 | rs4144829 | 17,903,654 | *LCORL/DCAF16* | C | T | 0.032 | 0.005 |
| 4 | rs6533183 | 106,133,184 | *TET2* | C | T | 0.008 | 0.004 |
| 4 | rs2715026 | 120,616,805 | *PDE5A* | C | G | 0.007 | 0.004 |
| 4 | rs116807401 | 135,121,721 | *PABPC4L* | C | T | 0.088 | 0.016 |
| 4 | rs6845999 | 145,565,826 | *LOC646576/HHIP* | T | C | 0.017 | 0.004 |
| 4 | rs4579095 | 174,726,635 | *NBLA00301* | A | G | 0.007 | 0.004 |
| 5 | rs12656216 | 36,160,668 | *SKP2* | A | G | 0.015 | 0.005 |
| 5 | rs1818782 | 39,424,628 | *DAB2* | C | A | 0.015 | 0.004 |
| 5 | rs351930 | 52,003,397 | *PELO* | T | A | 0.020 | 0.005 |
| 5 | rs854037 | 57,091,783 | *ACTBL2* | A | G | 0.020 | 0.005 |
| 5 | rs28365970 | 67,585,723 | *PIK3R1* | C | A | 0.015 | 0.005 |
| 5 | rs7709066 | 77,831,071 | *LHFPL2/ARSB* | C | T | 0.015 | 0.004 |
| 5 | rs1981627 | 133,838,180 | *PHF15* | G | A | 0.007 | 0.004 |
| 5 | rs11957761 | 141,058,487 | *ARAP3* | A | G | 0.009 | 0.005 |
| 5 | rs2946179 | 157,886,627 | *EBF1* | T | C | 0.004 | 0.005 |
| 5 | rs34471628 | 172,196,752 | *DUSP1* | G | A | 0.014 | 0.011 |
| 5 | rs4867699 | 172,748,540 | *STC2* | T | G | 0.016 | 0.004 |
| 6 | rs9379084 | 7,231,843 | *RREB1* | G | A | 0.004 | 0.006 |
| 6 | rs2747503 | 15,066,121 | *JARID2* | C | T | 0.013 | 0.005 |
| 6 | rs35261542 | 20,675,792 | *CDKAL1* | C | A | 0.049 | 0.005 |
| 6 | rs9379832 | 26,186,200 | *HIST1H2BE/HIST1H2BH* | A | G | 0.019 | 0.005 |
| 6 | rs113510833 | 29,671,067 | *HLA-F/ZFP57* | T | C | 0.000 | 0.007 |
| 6 | rs9366778 | 31,269,173 | *HLA-C* | G | A | 0.014 | 0.004 |
| 6 | rs6911024 | 31,368,451 | *MICA/HLA-C* | C | T | 0.002 | 0.007 |
| 6 | rs9267812 | 32,128,394 | *PPT2* | T | C | 0.015 | 0.006 |
| 6 | rs1547669 | 33,775,641 | *MLN* | G | A | 0.018 | 0.004 |
| 6 | rs75104038 | 34,190,104 | *HMGA1* | A | G | 0.024 | 0.009 |
| 6 | rs9348981 | 35,687,249 | *FKBP5/MAPK13/TEAD3* | T | G | 0.015 | 0.005 |
| 6 | rs2395668 | 37,105,893 | *PIM1* | A | G | 0.018 | 0.006 |
| 6 | rs7744700 | 53,349,401 | *GCLC* | T | A | 0.018 | 0.005 |
| 6 | rs185262229 | 84,105,136 | *ME1* | T | A | 0.091 | 0.023 |
| 6 | rs76094073 | 109,288,036 | *ARMC2/SESN1* | G | C | 0.011 | 0.006 |
| 6 | rs636252 | 117,157,774 | *GPRC6A/RFX6* | C | T | 0.001 | 0.004 |
| 6 | rs6925689 | 126,865,884 | *CENPW* | T | C | 0.018 | 0.004 |
| 6 | rs6569647 | 130,337,266 | *L3MBTL3* | T | C | 0.014 | 0.005 |
| 6 | rs6930558 | 141,878,920 | *NMBR* | T | G | 0.022 | 0.005 |
| 6 | rs962554 | 142,734,204 | *GPR126* | T | C | 0.015 | 0.005 |
| 6 | rs10872678 | 152,039,964 | *ESR1* | T | C | 0.028 | 0.005 |
| 6 | rs2934844 | 166,142,456 | *PDE10A* | T | A | 0.018 | 0.004 |
| 7 | rs4719648 | 2,756,832 | *AMZ1/GNA12* | C | T | 0.014 | 0.004 |
| 7 | rs59084784 | 22,739,562 | *IL6* | A | C | 0.011 | 0.004 |
| 7 | rs34776209 | 23,513,093 | *IGF2BP3* | C | T | 0.015 | 0.005 |
| 7 | rs2908279 | 44,174,857 | *MYL7/GCK* | T | G | 0.007 | 0.004 |
| 7 | rs2971669 | 44,231,778 | *GCK* | C | T | 0.003 | 0.005 |
| 7 | rs138715366 | 44,246,271 | *YKT6/GCK* | C | T | 0.235 | 0.022 |
| 7 | rs10265133 | 45,895,604 | *IGFBP1/IGFBP3* | T | G | 0.020 | 0.006 |
| 7 | rs11983722 | 46,298,647 | *IGFBP3* | A | T | 0.029 | 0.009 |
| 7 | rs10265057 | 47,275,737 | *TNS3* | G | A | 0.036 | 0.007 |
| 7 | rs2237467 | 50,733,316 | *GRB10* | A | G | 0.011 | 0.005 |
| 7 | rs112139215 | 73,034,559 | *MLXIPL* | A | C | 0.056 | 0.008 |
| 7 | rs2282978 | 92,264,410 | *CDK6* | C | T | 0.021 | 0.004 |
| 7 | rs45446698 | 99,332,948 | *CYP3A7-CYP3AP1* | T | G | 0.017 | 0.01 |
| 7 | rs6467157 | 127,660,763 | *SND1* | T | C | 0.014 | 0.005 |
| 7 | rs6958858 | 148,966,949 | *ZNF783* | C | T | 0.012 | 0.004 |
| 7 | rs3918226 | 150,690,176 | *NOS3* | T | C | 0.005 | 0.008 |
| 8 | rs62496903 | 6,446,938 | *MCPH1* | T | C | 0.028 | 0.008 |
| 8 | rs732563 | 23,345,526 | *ENTPD4/NKX3-1* | C | T | 0.019 | 0.004 |
| 8 | rs11778247 | 23,403,378 | *SLC25A37* | G | A | 0.000 | 0.006 |
| 8 | rs34036147 | 38,366,249 | *C8orf86/FGFR1* | T | C | 0.019 | 0.004 |
| 8 | rs116964396 | 41,505,849 | *NKX6-3* | A | C | 0.058 | 0.011 |
| 8 | rs13266210 | 41,533,514 | *ANK1* | A | G | 0.030 | 0.005 |
| 8 | rs72656010 | 57,122,215 | *PLAG1* | T | C | 0.026 | 0.006 |
| 8 | rs6995390 | 77,611,012 | *ZFHX4* | A | T | 0.014 | 0.006 |
| 8 | rs7819593 | 106,115,172 | *ZFPM2* | C | T | 0.023 | 0.005 |
| 8 | rs10283100 | 120,596,023 | *ENPP2* | G | A | 0.033 | 0.009 |
| 8 | rs13271368 | 126,506,140 | *TRIB1* | C | T | 0.021 | 0.005 |
| 8 | rs2608029 | 129,170,126 | *MIR1208* | G | C | 0.011 | 0.004 |
| 8 | rs13257363 | 142,252,580 | *SLC45A4* | G | A | 0.017 | 0.004 |
| 8 | rs9657468 | 142,362,391 | *GPR20* | G | T | 0.018 | 0.004 |
| 9 | rs10814916 | 4,293,150 | *GLIS3* | A | C | 0.018 | 0.004 |
| 9 | rs62562580 | 94,252,219 | *NFIL3/ROR2* | G | C | 0.011 | 0.005 |
| 9 | rs7854962 | 96,900,505 | *PTPDC1* | C | G | 0.016 | 0.005 |
| 9 | rs28457693 | 98,217,348 | *PTCH1/FANCC* | G | A | 0.040 | 0.007 |
| 9 | rs2418135 | 113,901,309 | *LPAR1* | A | G | 0.012 | 0.004 |
| 9 | rs72760655 | 116,916,214 | *COL27A1* | A | C | 0.009 | 0.004 |
| 9 | rs1323438 | 119,115,531 | *PAPPA* | C | T | 0.020 | 0.005 |
| 9 | rs3933326 | 123,633,948 | *PHF19* | G | A | 0.023 | 0.004 |
| 9 | rs10985827 | 125,701,608 | *RABGAP1/GPR21* | G | T | 0.027 | 0.006 |
| 9 | rs28505901 | 139,241,030 | *GPSM1* | A | G | 0.024 | 0.005 |
| 10 | rs6602476 | 11,122,635 | *CELF2* | C | A | 0.001 | 0.005 |
| 10 | rs4350272 | 25,056,118 | *ARHGAP21* | A | G | 0.017 | 0.005 |
| 10 | rs9645500 | 70,986,723 | *HKDC1/HK1* | G | T | 0.019 | 0.004 |
| 10 | rs7075355 | 82,208,878 | *TSPAN14* | A | G | 0.010 | 0.004 |
| 10 | rs558443 | 90,013,195 | *RNLS/PTEN* | A | C | 0.019 | 0.005 |
| 10 | rs1112718 | 94,479,107 | *HHEX/IDE* | G | A | 0.036 | 0.004 |
| 10 | rs2274224 | 96,039,597 | *PLCE1* | C | G | 0.019 | 0.004 |
| 10 | rs562974282 | 104,201,070 | *MIR146B* | T | G | 0.126 | 0.054 |
| 10 | rs10883846 | 104,958,244 | *NT5C2/CYP17A1* | C | T | 0.016 | 0.004 |
| 10 | rs1967840 | 112,026,082 | *MXI1* | A | G | 0.023 | 0.005 |
| 10 | rs7903146 | 114,758,349 | *TCF7L2* | T | C | 0.003 | 0.005 |
| 10 | rs7076938 | 115,789,375 | *ADRB1* | T | C | 0.029 | 0.005 |
| 10 | rs71486610 | 124,134,803 | *PLEKHA1* | C | G | 0.016 | 0.004 |
| 11 | rs11042596 | 2,118,860 | *INS-IGF2* | T | G | 0.027 | 0.004 |
| 11 | rs151216 | 2,680,815 | *KCNQ1/CDKN1C* | C | T | 0.021 | 0.007 |
| 11 | rs234864 | 2,857,297 | *KCNQ1* | A | G | 0.017 | 0.004 |
| 11 | rs2168101 | 8,255,408 | *LMO1* | A | C | 0.015 | 0.005 |
| 11 | rs4444073 | 10,331,664 | *ADM* | A | C | 0.023 | 0.004 |
| 11 | rs5030317 | 32,410,337 | *WT1* | C | G | 0.007 | 0.005 |
| 11 | rs10437653 | 46,297,631 | *CREB3L1* | A | C | 0.002 | 0.004 |
| 11 | rs10734564 | 48,160,429 | *PTPRJ* | G | A | 0.009 | 0.005 |
| 11 | rs12802960 | 58,174,775 | *OR5B3* | C | T | 0.014 | 0.005 |
| 11 | rs3765041 | 64,127,230 | *RPS6KA4* | G | T | 0.004 | 0.005 |
| 11 | rs7102454 | 65,594,820 | *SNX32/EFEMP2* | C | T | 0.012 | 0.004 |
| 11 | rs667515 | 69,449,076 | *CCND1* | G | C | 0.013 | 0.004 |
| 11 | rs61885091 | 69,791,952 | *ANO1/FGF4* | A | G | 0.024 | 0.006 |
| 11 | rs10830963* | 92,708,710 | *MTNR1B* | C | G | 0.002 | 0.005 |
| 11 | rs10895278 | 102,095,335 | *YAP1* | T | C | 0.001 | 0.004 |
| 11 | rs57414412 | 111,769,431 | *CRYAB* | G | A | 0.013 | 0.005 |
| 12 | rs76895963 | 4,384,844 | *CCND2* | G | T | 0.051 | 0.016 |
| 12 | rs11055030 | 12,878,349 | *APOLD1* | G | C | 0.022 | 0.005 |
| 12 | rs118106744 | 21,936,398 | *KCNJ8* | C | T | 0.038 | 0.009 |
| 12 | rs2307024 | 22,005,003 | *ABCC9* | T | G | 0.000 | 0.004 |
| 12 | rs2306547 | 26,877,885 | *ITPR2* | C | T | 0.016 | 0.004 |
| 12 | rs11051061 | 30,914,668 | *CAPRIN2* | A | G | 0.001 | 0.005 |
| 12 | rs6582623 | 46,613,394 | *SLC38A1* | C | T | 0.020 | 0.006 |
| 12 | rs180438 | 47,187,260 | *SLC38A4* | A | G | 0.007 | 0.005 |
| 12 | rs145775785 | 65,902,265 | *MSRB3* | T | C | 0.066 | 0.018 |
| 12 | rs7968682 | 66,371,880 | *HMGA2* | G | T | 0.037 | 0.004 |
| 12 | rs1533688 | 102,772,745 | *IGF1* | T | C | 0.004 | 0.005 |
| 12 | rs2647873 | 103,081,192 | *LINC00485/IGF1* | A | G | 0.009 | 0.004 |
| 12 | rs3184504 | 111,884,608 | *SH2B3* | C | T | 0.005 | 0.004 |
| 12 | rs80019595 | 121,417,306 | *HNF1A* | T | C | 0.036 | 0.013 |
| 12 | rs139429176 | 121,632,160 | *P2RX7/HNF1A* | C | T | 0.063 | 0.02 |
| 13 | rs55836809 | 28,502,874 | *PDX1* | A | G | 0.023 | 0.005 |
| 13 | rs9508017 | 28,934,364 | *FLT1* | C | T | 0.018 | 0.005 |
| 13 | rs7983505 | 33,557,173 | *KL* | T | A | 0.017 | 0.006 |
| 13 | rs9549046 | 40,647,206 | *LINC00332* | A | G | 0.027 | 0.006 |
| 13 | rs34217484 | 48,854,550 | *LINC00441/RB1* | A | T | 0.012 | 0.005 |
| 13 | rs9318511 | 78,601,413 | *LINC00446* | C | A | 0.024 | 0.006 |
| 13 | rs2262207 | 114,136,110 | *DCUN1D2* | A | G | 0.018 | 0.005 |
| 14 | rs10147938 | 31,885,951 | *HEATR5A* | T | C | 0.017 | 0.004 |
| 14 | rs72681869 | 50,655,357 | *SOS2* | C | G | 0.108 | 0.021 |
| 14 | rs12896104 | 74,306,014 | *PTGR2* | G | A | 0.016 | 0.004 |
| 14 | rs6575803 | 101,257,755 | *MIR2392/DLK1* | C | T | 0.034 | 0.007 |
| 15 | rs75844534 | 38,667,117 | *SPRED1* | A | C | 0.036 | 0.006 |
| 15 | rs2928148 | 41,401,550 | *INO80* | G | A | 0.004 | 0.004 |
| 15 | rs62023486 | 53,070,589 | *ONECUT1* | A | G | 0.016 | 0.007 |
| 15 | rs339969 | 60,883,281 | *RORA* | A | C | 0.011 | 0.004 |
| 15 | rs2118611 | 67,401,466 | *SMAD3* | C | T | 0.011 | 0.005 |
| 15 | rs5742915 | 74,336,633 | *PML* | C | T | 0.011 | 0.004 |
| 15 | rs3784789 | 75,082,552 | *CSK* | C | G | 0.018 | 0.004 |
| 15 | rs8038207 | 86,316,570 | *KLHL25/AKAP13* | G | T | 0.003 | 0.004 |
| 15 | rs12443252 | 91,064,690 | *CRTC3* | C | T | 0.007 | 0.004 |
| 15 | rs4932373 | 91,429,287 | *FES/FURIN* | A | C | 0.010 | 0.004 |
| 15 | rs55958435 | 96,852,638 | *NR2F2* | A | G | 0.022 | 0.005 |
| 15 | rs7402983 | 99,193,276 | *IGF1R* | A | C | 0.027 | 0.004 |
| 16 | rs2238464 | 2,332,577 | *ABCA3* | T | C | 0.016 | 0.004 |
| 16 | rs2045457 | 20,046,115 | *GPR139/GPRC5B* | G | A | 0.012 | 0.004 |
| 16 | rs12446550 | 28,543,381 | *NUPR1/APOBR* | G | A | 0.006 | 0.004 |
| 16 | rs7205514 | 50,271,806 | *PAPD5* | T | G | 0.018 | 0.005 |
| 16 | rs40434 | 55,699,525 | *SLC6A2* | G | A | 0.017 | 0.004 |
| 16 | rs11646700 | 68,421,668 | *SMPD3/PRMT7* | G | A | 0.013 | 0.004 |
| 16 | rs35549608 | 72,227,398 | *PMFBP1* | T | C | 0.011 | 0.006 |
| 16 | rs11641308 | 75,312,023 | *BCAR1* | C | T | 0.005 | 0.004 |
| 16 | rs9783782 | 88,317,285 | *ZNF469* | G | T | 0.010 | 0.005 |
| 17 | rs222857 | 7,164,563 | *CLDN7/SLC2A4* | T | C | 0.026 | 0.004 |
| 17 | rs4511593 | 7,455,536 | *TNFSF12-TNFSF13* | T | C | 0.019 | 0.004 |
| 17 | rs78378222 | 7,571,752 | *TP53* | G | T | 0.058 | 0.019 |
| 17 | rs1242516 | 17,387,079 | *MED9* | C | T | 0.016 | 0.006 |
| 17 | rs34717629 | 17,610,404 | *RAI1* | G | A | 0.015 | 0.005 |
| 17 | rs9909342 | 25,652,275 | *WSB1* | A | G | 0.019 | 0.004 |
| 17 | rs7223535 | 29,211,667 | *ATAD5* | G | A | 0.020 | 0.005 |
| 17 | rs4794720 | 55,392,223 | *MSI2* | G | A | 0.013 | 0.004 |
| 17 | rs11867479 | 68,090,207 | *KCNJ16* | T | C | 0.018 | 0.004 |
| 17 | rs10221267 | 68,464,662 | *KCNJ2* | T | C | 0.018 | 0.004 |
| 17 | rs73354194 | 79,905,947 | *MYADML2* | C | T | 0.060 | 0.014 |
| 17 | rs9912553 | 79,959,703 | *ASPSCR1* | G | C | 0.006 | 0.005 |
| 18 | rs11082304 | 20,720,973 | *CABLES1* | T | G | 0.013 | 0.004 |
| 18 | rs2663842 | 55,449,516 | *ATP8B1* | A | G | 0.019 | 0.004 |
| 19 | rs2779165 | 4,915,447 | *UHRF1* | G | C | 0.018 | 0.005 |
| 19 | rs8106042 | 7,161,849 | *INSR* | G | C | 0.023 | 0.005 |
| 19 | rs2967677 | 8,789,721 | *ACTL9* | T | C | 0.004 | 0.006 |
| 19 | rs11085720 | 10,317,763 | *DNMT1* | A | G | 0.007 | 0.004 |
| 19 | rs41355649 | 33,790,556 | *CEBPA* | G | A | 0.042 | 0.008 |
| 19 | rs1129156 | 40,719,076 | *MAP3K10/AKT2* | T | C | 0.022 | 0.005 |
| 19 | rs147957154 | 43,431,040 | *PSG7* | T | C | 0.026 | 0.006 |
| 19 | rs516246 | 49,206,172 | *FUT2* | C | T | 0.017 | 0.004 |
| 19 | rs255773 | 54,723,546 | *LILRB3/RPS9* | C | T | 0.018 | 0.004 |
| 19 | rs147110934 | 55,993,436 | *ZNF628* | G | T | 0.055 | 0.014 |
| 19 | rs12461110 | 56,320,663 | *NLRP11* | G | A | 0.005 | 0.004 |
| 19 | rs304001 | 56,423,668 | *NLRP13* | A | G | 0.003 | 0.004 |
| 20 | rs1964859 | 607,805 | *TCF15* | T | C | 0.020 | 0.005 |
| 20 | rs6040076 | 10,658,882 | *JAG1* | C | G | 0.015 | 0.004 |
| 20 | rs6033062 | 11,207,419 | *LOC339593* | A | T | 0.014 | 0.004 |
| 20 | rs1203876 | 22,540,915 | *LINC00261/FOXA2* | C | A | 0.055 | 0.01 |
| 20 | rs11698914 | 31,327,144 | *COMMD7* | C | G | 0.029 | 0.005 |
| 20 | rs181451002 | 32,466,219 | *CHMP4B* | A | G | 0.006 | 0.014 |
| 20 | rs2889874 | 33,715,777 | *EDEM2/MYH7B* | G | T | 0.014 | 0.004 |
| 20 | rs1012167 | 39,159,119 | *MAFB* | C | T | 0.024 | 0.004 |
| 20 | rs753381 | 39,797,465 | *PLCG1* | T | C | 0.018 | 0.004 |
| 20 | rs4809731 | 47,495,767 | *ARFGEF2* | G | C | 0.015 | 0.006 |
| 20 | rs6026449 | 57,272,617 | *STX16-NPEPL1/GNAS* | C | T | 0.018 | 0.004 |
| 20 | rs73143584 | 62,445,702 | *ZBTB46* | A | G | 0.031 | 0.007 |
| 20 | rs817329 | 62,597,694 | *ZNF512B* | T | G | 0.012 | 0.004 |
| 21 | rs2229742 | 16,339,172 | *NRIP1* | G | C | 0.028 | 0.006 |
| 21 | rs75518158 | 38,393,567 | *DSCR6* | C | A | 0.046 | 0.011 |
| 21 | rs220193 | 43,581,308 | *UMODL1* | A | G | 0.018 | 0.005 |
| 22 | rs134594 | 29,468,456 | *KREMEN1* | C | T | 0.022 | 0.004 |
| 22 | rs5750561 | 38,595,260 | *MAFF* | A | T | 0.011 | 0.004 |
| 22 | rs41311445 | 42,070,374 | *NHP2L1/SREBF2* | A | C | 0.034 | 0.007 |
| 22 | rs11704481 | 45,732,328 | *FAM118A* | G | A | 0.013 | 0.004 |
| 22 | rs7285579 | 46,441,980 | *LOC100271722* | C | T | 0.018 | 0.005 |
| 22 | rs28681372 | 50,351,977 | *PIM3* | A | G | 0.019 | 0.004 |

Table S3. Summary information of the SEM-adjusted maternal effects for the instrumental variables of birth weight.

| CHR | SNP | Position (hg19) | Gene | Effect Allele | Other Allele | Beta | Se |
| --- | --- | --- | --- | --- | --- | --- | --- |
| 1 | rs17367504 | 11,862,778 | *MTHFR* | G | A | 0.032 | 0.006 |
| 1 | rs2807319 | 22,554,176 | *WNT4* | A | G | 0.004 | 0.008 |
| 1 | rs4908404 | 28,691,734 | *PHACTR4* | C | T | 0.020 | 0.005 |
| 1 | rs12401656 | 43,456,767 | *FLJ32224/SLC2A1* | A | G | 0.006 | 0.007 |
| 1 | rs1937436 | 66,441,329 | *PDE4B* | G | A | 0.003 | 0.005 |
| 1 | rs2755253 | 67,470,843 | *SLC35D1/WDR78* | C | T | 0.012 | 0.005 |
| 1 | rs787541 | 68,051,230 | *GADD45A* | C | G | 0.020 | 0.005 |
| 1 | rs7525870 | 78,269,207 | *FAM73A* | G | A | 0.004 | 0.005 |
| 1 | rs41276588 | 118,148,384 | *FAM46C* | A | G | 0.017 | 0.005 |
| 1 | rs80278614 | 119,412,317 | *TBX15* | G | A | 0.014 | 0.011 |
| 1 | rs116276359 | 151,821,430 | *THEM5* | C | A | 0.015 | 0.014 |
| 1 | rs6426985 | 154,813,619 | *DCST2/KCNN3* | A | G | 0.011 | 0.005 |
| 1 | rs670523 | 155,878,732 | *RIT1/LMNA* | G | A | 0.005 | 0.005 |
| 1 | rs144126567 | 161,510,516 | *FCGR3A* | C | G | 0.005 | 0.009 |
| 1 | rs67775399 | 161,572,353 | *FCGR2B/FCGR2C/HSPA6* | C | T | 0.016 | 0.006 |
| 1 | rs10913200 | 176,521,655 | *PAPPA2* | G | A | 0.029 | 0.014 |
| 1 | rs61830764 | 212,289,976 | *DTL* | G | A | 0.003 | 0.005 |
| 1 | rs7541039 | 214,176,779 | *PROX1* | C | T | 0.009 | 0.005 |
| 1 | rs3806315 | 214,724,668 | *PTPN14* | A | G | 0.006 | 0.005 |
| 1 | rs4655413 | 215,828,499 | *USH2A* | T | C | 0.004 | 0.005 |
| 1 | rs708122 | 228,216,997 | *WNT3A* | C | A | 0.004 | 0.005 |
| 2 | rs11893688 | 9,695,282 | *ADAM17* | T | C | 0.013 | 0.005 |
| 2 | rs2551347 | 23,912,401 | *KLHL29* | C | T | 0.006 | 0.005 |
| 2 | rs1179494 | 36,809,496 | *FEZ2* | G | C | 0.017 | 0.005 |
| 2 | rs754868 | 43,185,532 | *HAAO* | A | G | 0.004 | 0.005 |
| 2 | rs4952673 | 43,423,870 | *ZFP36L2* | A | G | 0.025 | 0.005 |
| 2 | rs17034876 | 46,484,310 | *EPAS1* | T | C | 0.011 | 0.005 |
| 2 | rs7596521 | 46,918,665 | *SOCS5* | G | A | 0.023 | 0.005 |
| 2 | rs186606513 | 97,482,001 | *CNNM3* | G | A | 0.030 | 0.020 |
| 2 | rs12104672 | 109,151,173 | *LIMS1* | G | T | 0.004 | 0.005 |
| 2 | rs11680809 | 113,570,809 | *IL1B* | C | A | 0.010 | 0.005 |
| 2 | rs12623454 | 121,326,297 | *LOC84931* | C | G | 0.003 | 0.005 |
| 2 | rs56188432 | 158,406,865 | *ACVR1C* | G | A | 0.041 | 0.050 |
| 2 | rs10173538 | 160,569,276 | *7-Mar* | C | T | 0.006 | 0.005 |
| 2 | rs560887 | 169,763,148 | *G6PC2* | C | T | 0.038 | 0.005 |
| 2 | rs139557015 | 182,072,326 | *MIR4437* | T | C | 0.066 | 0.013 |
| 2 | rs2280235 | 191,843,830 | *STAT1* | G | A | 0.005 | 0.005 |
| 2 | rs10181515 | 227,019,461 | *LOC646736/COL4A4/IRS1* | C | T | 0.005 | 0.006 |
| 3 | rs2574727 | 11,636,508 | *VGLL4* | G | A | 0.042 | 0.011 |
| 3 | rs9855896 | 14,287,150 | *LSM3* | G | A | 0.033 | 0.006 |
| 3 | rs60573957 | 14,884,409 | *FGD5* | T | C | 0.018 | 0.005 |
| 3 | rs2242116 | 46,941,116 | *PTH1R* | A | G | 0.012 | 0.005 |
| 3 | rs3965156 | 66,484,956 | *LRIG1* | C | A | 0.003 | 0.005 |
| 3 | rs73226528 | 113,398,642 | *KIAA2018* | C | T | 0.024 | 0.006 |
| 3 | rs9851257 | 123,125,711 | *ADCY5* | T | A | 0.027 | 0.005 |
| 3 | rs17315501 | 139,029,676 | *MRPS22* | G | A | 0.076 | 0.018 |
| 3 | rs6440006 | 141,142,691 | *ZBTB38* | A | G | 0.020 | 0.005 |
| 3 | rs2306700 | 142,123,841 | *XRN1* | T | C | 0.006 | 0.007 |
| 3 | rs10935733 | 148,622,968 | *CPA3/AGTR1* | C | T | 0.001 | 0.005 |
| 3 | rs4679760 | 155,855,418 | *KCNAB1* | G | C | 0.038 | 0.005 |
| 3 | rs1482852 | 156,798,294 | *LOC339894/CCNL1* | G | A | 0.003 | 0.005 |
| 3 | rs11711420 | 183,349,010 | *KLHL24* | G | T | 0.006 | 0.005 |
| 3 | rs11545169 | 184,020,542 | *PSMD2* | G | T | 0.003 | 0.006 |
| 4 | rs2174633 | 17,917,781 | *LCORL/DCAF16* | A | C | 0.011 | 0.005 |
| 4 | rs2189234 | 106,075,498 | *TET2* | G | T | 0.026 | 0.005 |
| 4 | rs2715026 | 120,616,805 | *PDE5A* | G | C | 0.022 | 0.005 |
| 4 | rs116807401 | 135,121,721 | *PABPC4L* | T | C | 0.016 | 0.018 |
| 4 | rs2131354 | 145,599,908 | *LOC646576/HHIP* | A | G | 0.019 | 0.005 |
| 4 | rs4579095 | 174,726,635 | *NBLA00301* | G | A | 0.023 | 0.005 |
| 5 | rs12656216 | 36,160,668 | *SKP2* | A | G | 0.004 | 0.006 |
| 5 | rs1818782 | 39,424,628 | *DAB2* | A | C | 0.001 | 0.005 |
| 5 | rs351930 | 52,003,397 | *PELO* | A | T | 0.005 | 0.006 |
| 5 | rs854037 | 57,091,783 | *ACTBL2* | A | G | 0.011 | 0.006 |
| 5 | rs28365970 | 67,585,723 | *PIK3R1* | C | A | 0.010 | 0.005 |
| 5 | rs7709066 | 77,831,071 | *LHFPL2/ARSB* | T | C | 0.001 | 0.005 |
| 5 | rs6871635 | 133,830,395 | *PHF15* | G | A | 0.022 | 0.005 |
| 5 | rs11957761 | 141,058,487 | *ARAP3* | G | A | 0.025 | 0.006 |
| 5 | rs2946179 | 157,886,627 | *EBF1* | C | T | 0.045 | 0.005 |
| 5 | rs34471628 | 172,196,752 | *DUSP1* | A | G | 0.067 | 0.012 |
| 5 | rs4867699 | 172,748,540 | *STC2* | G | T | 0.001 | 0.005 |
| 6 | rs9379084 | 7,231,843 | *RREB1* | G | A | 0.040 | 0.007 |
| 6 | rs2747503 | 15,066,121 | *JARID2* | C | T | 0.001 | 0.005 |
| 6 | rs35261542 | 20,675,792 | *CDKAL1* | A | C | 0.019 | 0.005 |
| 6 | rs809871 | 26,256,526 | *HIST1H2BE/HIST1H2BH* | C | G | 0.015 | 0.005 |
| 6 | rs113510833 | 29,671,067 | *HLA-F/ZFP57* | T | C | 0.028 | 0.008 |
| 6 | rs9366778 | 31,269,173 | *HLA-C* | G | A | 0.005 | 0.005 |
| 6 | rs6911024 | 31,368,451 | *MICA/HLA-C* | T | C | 0.040 | 0.008 |
| 6 | rs9267812 | 32,128,394 | *PPT2* | T | C | 0.014 | 0.007 |
| 6 | rs1547669 | 33,775,641 | *MLN* | A | G | 0.002 | 0.005 |
| 6 | rs75034466 | 34,199,815 | *HMGA1* | T | C | 0.051 | 0.011 |
| 6 | rs6911621 | 35,529,025 | *FKBP5/MAPK13/TEAD3* | T | C | 0.022 | 0.005 |
| 6 | rs2395668 | 37,105,893 | *PIM1* | A | G | 0.002 | 0.006 |
| 6 | rs7744700 | 53,349,401 | *GCLC* | T | A | 0.001 | 0.005 |
| 6 | rs185262229 | 84,105,136 | *ME1* | A | T | 0.028 | 0.027 |
| 6 | rs6568554 | 109,290,319 | *ARMC2/SESN1* | A | C | 0.025 | 0.007 |
| 6 | rs636252 | 117,157,774 | *GPRC6A/RFX6* | T | C | 0.017 | 0.005 |
| 6 | rs6925689 | 126,865,884 | *CENPW* | C | T | 0.008 | 0.005 |
| 6 | rs1415701 | 130,345,835 | *L3MBTL3* | G | A | 0.019 | 0.005 |
| 6 | rs6930558 | 141,878,920 | *NMBR* | T | G | 0.000 | 0.005 |
| 6 | rs4518515 | 142,584,429 | *GPR126* | A | T | 0.017 | 0.005 |
| 6 | rs7772579 | 152,042,502 | *ESR1* | A | C | 0.006 | 0.005 |
| 6 | rs2934844 | 166,142,456 | *PDE10A* | T | A | 0.000 | 0.005 |
| 7 | rs1724889 | 2,741,021 | *AMZ1/GNA12* | G | A | 0.017 | 0.006 |
| 7 | rs7808457 | 22,798,265 | *IL6* | A | T | 0.017 | 0.005 |
| 7 | rs34776209 | 23,513,093 | *IGF2BP3* | C | T | 0.014 | 0.006 |
| 7 | rs2908279 | 44,174,857 | *MYL7/GCK* | T | G | 0.010 | 0.005 |
| 7 | rs2971669 | 44,231,778 | *GCK* | T | C | 0.028 | 0.006 |
| 7 | rs138715366 | 44,246,271 | *YKT6/GCK* | C | T | 0.001 | 0.026 |
| 7 | rs10265133 | 45,895,604 | *IGFBP1/IGFBP3* | G | T | 0.034 | 0.007 |
| 7 | rs11983722 | 46,298,647 | *IGFBP3* | A | T | 0.000 | 0.009 |
| 7 | rs10265057 | 47,275,737 | *TNS3* | A | G | 0.014 | 0.008 |
| 7 | rs2237467 | 50,733,316 | *GRB10* | A | G | 0.012 | 0.006 |
| 7 | rs112139215 | 73,034,559 | *MLXIPL* | C | A | 0.010 | 0.009 |
| 7 | rs2282978 | 92,264,410 | *CDK6* | T | C | 0.003 | 0.005 |
| 7 | rs45446698 | 99,332,948 | *CYP3A7-CYP3AP1* | G | T | 0.077 | 0.012 |
| 7 | rs13231367 | 127,509,070 | *SND1* | G | A | 0.018 | 0.005 |
| 7 | rs6958858 | 148,966,949 | *ZNF783* | C | T | 0.004 | 0.005 |
| 7 | rs3918226 | 150,690,176 | *NOS3* | C | T | 0.040 | 0.009 |
| 8 | rs62496903 | 6,446,938 | *MCPH1* | T | C | 0.003 | 0.009 |
| 8 | rs732563 | 23,345,526 | *ENTPD4/NKX3-1* | T | C | 0.002 | 0.005 |
| 8 | rs11778247 | 23,403,378 | *SLC25A37* | G | A | 0.026 | 0.006 |
| 8 | rs34036147 | 38,366,249 | *C8orf86/FGFR1* | C | T | 0.002 | 0.005 |
| 8 | rs116964396 | 41,505,849 | *NKX6-3* | C | A | 0.031 | 0.013 |
| 8 | rs13266210 | 41,533,514 | *ANK1* | G | A | 0.005 | 0.006 |
| 8 | rs72656010 | 57,122,215 | *PLAG1* | C | T | 0.001 | 0.007 |
| 8 | rs6995390 | 77,611,012 | *ZFHX4* | T | A | 0.039 | 0.006 |
| 8 | rs7819593 | 106,115,172 | *ZFPM2* | T | C | 0.002 | 0.005 |
| 8 | rs10283100 | 120,596,023 | *ENPP2* | G | A | 0.015 | 0.010 |
| 8 | rs13271368 | 126,506,140 | *TRIB1* | C | T | 0.002 | 0.006 |
| 8 | rs2608029 | 129,170,126 | *MIR1208* | G | C | 0.009 | 0.005 |
| 8 | rs13257363 | 142,252,580 | *SLC45A4* | G | A | 0.002 | 0.005 |
| 8 | rs9657468 | 142,362,391 | *GPR20* | T | G | 0.005 | 0.005 |
| 9 | rs10814916 | 4,293,150 | *GLIS3* | C | A | 0.025 | 0.005 |
| 9 | rs62562580 | 94,252,219 | *NFIL3/ROR2* | G | C | 0.010 | 0.006 |
| 9 | rs7854962 | 96,900,505 | *PTPDC1* | C | G | 0.010 | 0.006 |
| 9 | rs28457693 | 98,217,348 | *PTCH1/FANCC* | G | A | 0.009 | 0.008 |
| 9 | rs1411424 | 113,892,963 | *LPAR1* | A | G | 0.016 | 0.005 |
| 9 | rs72760655 | 116,916,214 | *COL27A1* | C | A | 0.031 | 0.005 |
| 9 | rs1323438 | 119,115,531 | *PAPPA* | T | C | 0.001 | 0.005 |
| 9 | rs3933326 | 123,633,948 | *PHF19* | A | G | 0.001 | 0.005 |
| 9 | rs10985827 | 125,701,608 | *RABGAP1/GPR21* | G | T | 0.004 | 0.007 |
| 9 | rs28505901 | 139,241,030 | *GPSM1* | A | G | 0.001 | 0.006 |
| 10 | rs6602476 | 11,122,635 | *CELF2* | A | C | 0.016 | 0.005 |
| 10 | rs4350272 | 25,056,118 | *ARHGAP21* | A | G | 0.001 | 0.005 |
| 10 | rs5030938 | 70,975,916 | *HKDC1/HK1* | T | C | 0.010 | 0.005 |
| 10 | rs7075355 | 82,208,878 | *TSPAN14* | A | G | 0.009 | 0.005 |
| 10 | rs558443 | 90,013,195 | *RNLS/PTEN* | C | A | 0.001 | 0.006 |
| 10 | rs1112718 | 94,479,107 | *HHEX/IDE* | A | G | 0.018 | 0.005 |
| 10 | rs3740360 | 96,025,491 | *PLCE1* | C | A | 0.044 | 0.007 |
| 10 | rs562974282 | 104,201,070 | *MIR146B* | G | T | 0.269 | 0.055 |
| 10 | rs10883846 | 104,958,244 | *NT5C2/CYP17A1* | C | T | 0.003 | 0.005 |
| 10 | rs1967840 | 112,026,082 | *MXI1* | G | A | 0.005 | 0.006 |
| 10 | rs7903146 | 114,758,349 | *TCF7L2* | T | C | 0.020 | 0.005 |
| 10 | rs1801253 | 115,805,056 | *ADRB1* | C | G | 0.010 | 0.005 |
| 10 | rs2292626 | 124,186,714 | *PLEKHA1* | T | C | 0.011 | 0.005 |
| 11 | rs11042596 | 2,118,860 | *INS-IGF2* | G | T | 0.007 | 0.005 |
| 11 | rs151216 | 2,680,815 | *KCNQ1/CDKN1C* | C | T | 0.007 | 0.008 |
| 11 | rs231350 | 2,713,649 | *KCNQ1* | C | A | 0.016 | 0.005 |
| 11 | rs2168101 | 8,255,408 | *LMO1* | C | A | 0.039 | 0.005 |
| 11 | rs4444073 | 10,331,664 | *ADM* | C | A | 0.005 | 0.005 |
| 11 | rs12574749 | 32,405,355 | *WT1* | C | A | 0.022 | 0.005 |
| 11 | rs10437653 | 46,297,631 | *CREB3L1* | A | C | 0.018 | 0.005 |
| 11 | rs10734564 | 48,160,429 | *PTPRJ* | A | G | 0.033 | 0.006 |
| 11 | rs12802960 | 58,174,775 | *OR5B3* | C | T | 0.007 | 0.006 |
| 11 | rs3765041 | 64,127,230 | *RPS6KA4* | T | G | 0.017 | 0.005 |
| 11 | rs7102454 | 65,594,820 | *SNX32/EFEMP2* | C | T | 0.001 | 0.005 |
| 11 | rs667515 | 69,449,076 | *CCND1* | G | C | 0.011 | 0.005 |
| 11 | rs61885091 | 69,791,952 | *ANO1/FGF4* | A | G | 0.004 | 0.006 |
| 11 | rs10830963* | 92,708,710 | *MTNR1B* | G | C | 0.046 | 0.005 |
| 11 | rs10895278 | 102,095,335 | *YAP1* | C | T | 0.025 | 0.005 |
| 11 | rs57414412 | 111,769,431 | *CRYAB* | G | A | 0.003 | 0.005 |
| 12 | rs76895963 | 4,384,844 | *CCND2* | G | T | 0.040 | 0.018 |
| 12 | rs11055030 | 12,878,349 | *APOLD1* | C | G | 0.005 | 0.005 |
| 12 | rs118106744 | 21,936,398 | *KCNJ8* | T | C | 0.010 | 0.011 |
| 12 | rs2307024 | 22,005,003 | *ABCC9* | T | G | 0.014 | 0.005 |
| 12 | rs895964 | 26,858,066 | *ITPR2* | G | A | 0.010 | 0.005 |
| 12 | rs11051061 | 30,914,668 | *CAPRIN2* | A | G | 0.026 | 0.005 |
| 12 | rs6582623 | 46,613,394 | *SLC38A1* | C | T | 0.011 | 0.007 |
| 12 | rs180438 | 47,187,260 | *SLC38A4* | G | A | 0.039 | 0.006 |
| 12 | rs145775785 | 65,902,265 | *MSRB3* | T | C | 0.000 | 0.020 |
| 12 | rs8756 | 66,359,752 | *HMGA2* | C | A | 0.009 | 0.005 |
| 12 | rs1533688 | 102,772,745 | *IGF1* | C | T | 0.025 | 0.006 |
| 12 | rs17033114 | 103,123,339 | *LINC00485/IGF1* | T | C | 0.053 | 0.010 |
| 12 | rs3184504 | 111,884,608 | *SH2B3* | C | T | 0.034 | 0.005 |
| 12 | rs80019595 | 121,417,306 | *HNF1A* | T | C | 0.014 | 0.015 |
| 12 | rs139429176 | 121,632,160 | *P2RX7/HNF1A* | C | T | 0.018 | 0.022 |
| 13 | rs55836809 | 28,502,874 | *PDX1* | G | A | 0.008 | 0.006 |
| 13 | rs9508017 | 28,934,364 | *FLT1* | T | C | 0.002 | 0.005 |
| 13 | rs7983505 | 33,557,173 | *KL* | T | A | 0.005 | 0.006 |
| 13 | rs9549046 | 40,647,206 | *LINC00332* | A | G | 0.001 | 0.008 |
| 13 | rs34217484 | 48,854,550 | *LINC00441/RB1* | A | T | 0.013 | 0.005 |
| 13 | rs9318511 | 78,601,413 | *LINC00446* | C | A | 0.007 | 0.007 |
| 13 | rs2262207 | 114,136,110 | *DCUN1D2* | A | G | 0.001 | 0.006 |
| 14 | rs10147938 | 31,885,951 | *HEATR5A* | C | T | 0.007 | 0.005 |
| 14 | rs72681869 | 50,655,357 | *SOS2* | G | C | 0.039 | 0.023 |
| 14 | rs12896104 | 74,306,014 | *PTGR2* | A | G | 0.005 | 0.005 |
| 14 | rs6575803 | 101,257,755 | *MIR2392/DLK1* | T | C | 0.005 | 0.008 |
| 15 | rs75844534 | 38,667,117 | *SPRED1* | C | A | 0.021 | 0.007 |
| 15 | rs2928148 | 41,401,550 | *INO80* | A | G | 0.020 | 0.005 |
| 15 | rs62023486 | 53,070,589 | *ONECUT1* | A | G | 0.015 | 0.008 |
| 15 | rs339969 | 60,883,281 | *RORA* | A | C | 0.010 | 0.005 |
| 15 | rs2118611 | 67,401,466 | *SMAD3* | T | C | 0.027 | 0.006 |
| 15 | rs5742915 | 74,336,633 | *PML* | C | T | 0.006 | 0.005 |
| 15 | rs3784789 | 75,082,552 | *CSK* | G | C | 0.030 | 0.005 |
| 15 | rs12909648 | 86,224,570 | *KLHL25/AKAP13* | G | A | 0.027 | 0.005 |
| 15 | rs12443252 | 91,064,690 | *CRTC3* | T | C | 0.023 | 0.005 |
| 15 | rs7183988 | 91,428,589 | *FES/FURIN* | G | T | 0.024 | 0.005 |
| 15 | rs55958435 | 96,852,638 | *NR2F2* | A | G | 0.004 | 0.006 |
| 15 | rs11630479 | 99,240,481 | *IGF1R* | G | A | 0.013 | 0.005 |
| 16 | rs2238464 | 2,332,577 | *ABCA3* | C | T | 0.002 | 0.005 |
| 16 | rs2045457 | 20,046,115 | *GPR139/GPRC5B* | G | A | 0.008 | 0.005 |
| 16 | rs12446550 | 28,543,381 | *NUPR1/APOBR* | A | G | 0.022 | 0.005 |
| 16 | rs7205514 | 50,271,806 | *PAPD5* | T | G | 0.000 | 0.005 |
| 16 | rs28544888 | 55,741,204 | *SLC6A2* | C | T | 0.002 | 0.008 |
| 16 | rs1868158 | 68,398,924 | *SMPD3/PRMT7* | T | C | 0.008 | 0.005 |
| 16 | rs35549608 | 72,227,398 | *PMFBP1* | C | T | 0.034 | 0.006 |
| 16 | rs11641308 | 75,312,023 | *BCAR1* | T | C | 0.023 | 0.005 |
| 16 | rs9783782 | 88,317,285 | *ZNF469* | G | T | 0.011 | 0.005 |
| 17 | rs2428362? | 7,180,274 | *CLDN7/SLC2A4* | T | C | 0.008 | 0.005 |
| 17 | rs4511593 | 7,455,536 | *TNFSF12-TNFSF13* | C | T | 0.004 | 0.005 |
| 17 | rs78378222 | 7,571,752 | *TP53* | G | T | 0.021 | 0.022 |
| 17 | rs1242516 | 17,387,079 | *MED9* | C | T | 0.006 | 0.007 |
| 17 | rs34717629 | 17,610,404 | *RAI1* | A | G | 0.032 | 0.006 |
| 17 | rs9909342 | 25,652,275 | *WSB1* | A | G | 0.001 | 0.005 |
| 17 | rs7223535 | 29,211,667 | *ATAD5* | G | A | 0.007 | 0.005 |
| 17 | rs4794720 | 55,392,223 | *MSI2* | G | A | 0.004 | 0.005 |
| 17 | rs11867479 | 68,090,207 | *KCNJ16* | C | T | 0.002 | 0.005 |
| 17 | rs10221267 | 68,464,662 | *KCNJ2* | C | T | 0.002 | 0.005 |
| 17 | rs73354194 | 79,905,947 | *MYADML2* | T | C | 0.011 | 0.016 |
| 17 | rs9912553 | 79,959,703 | *ASPSCR1* | G | C | 0.017 | 0.005 |
| 18 | rs11082304 | 20,720,973 | *CABLES1* | T | G | 0.003 | 0.005 |
| 18 | rs2663842 | 55,449,516 | *ATP8B1* | G | A | 0.006 | 0.005 |
| 19 | rs2779165 | 4,915,447 | *UHRF1* | G | C | 0.007 | 0.006 |
| 19 | rs8106042 | 7,161,849 | *INSR* | C | G | 0.006 | 0.005 |
| 19 | rs2967676 | 8,789,666 | *ACTL9* | A | C | 0.048 | 0.006 |
| 19 | rs11085720 | 10,317,763 | *DNMT1* | A | G | 0.012 | 0.005 |
| 19 | rs41355649 | 33,790,556 | *CEBPA* | A | G | 0.010 | 0.009 |
| 19 | rs1129156 | 40,719,076 | *MAP3K10/AKT2* | C | T | 0.007 | 0.005 |
| 19 | rs147957154 | 43,431,040 | *PSG7* | C | T | 0.012 | 0.007 |
| 19 | rs516246 | 49,206,172 | *FUT2* | C | T | 0.000 | 0.005 |
| 19 | rs255773 | 54,723,546 | *LILRB3/RPS9* | C | T | 0.004 | 0.005 |
| 19 | rs147110934 | 55,993,436 | *ZNF628* | G | T | 0.001 | 0.015 |
| 19 | rs12461110 | 56,320,663 | *NLRP11* | A | G | 0.022 | 0.005 |
| 19 | rs304001 | 56,423,668 | *NLRP13* | G | A | 0.023 | 0.005 |
| 20 | rs1964859 | 607,805 | *TCF15* | C | T | 0.006 | 0.005 |
| 20 | rs6040076 | 10,658,882 | *JAG1* | C | G | 0.009 | 0.005 |
| 20 | rs6033062 | 11,207,419 | *LOC339593* | A | T | 0.003 | 0.005 |
| 20 | rs1203876 | 22,540,915 | *LINC00261/FOXA2* | A | C | 0.040 | 0.011 |
| 20 | rs11698914 | 31,327,144 | *COMMD7* | C | G | 0.003 | 0.006 |
| 20 | rs181451002 | 32,466,219 | *CHMP4B* | G | A | 0.059 | 0.016 |
| 20 | rs3746448 | 33,572,979 | *EDEM2/MYH7B* | C | T | 0.022 | 0.006 |
| 20 | rs1012167 | 39,159,119 | *MAFB* | T | C | 0.003 | 0.005 |
| 20 | rs753381 | 39,797,465 | *PLCG1* | C | T | 0.007 | 0.005 |
| 20 | rs4809731 | 47,495,767 | *ARFGEF2* | G | C | 0.013 | 0.007 |
| 20 | rs6026449 | 57,272,617 | *STX16-NPEPL1/GNAS* | T | C | 0.006 | 0.005 |
| 20 | rs73143584 | 62,445,702 | *ZBTB46* | G | A | 0.007 | 0.008 |
| 20 | rs817329 | 62,597,694 | *ZNF512B* | T | G | 0.011 | 0.005 |
| 21 | rs2229742 | 16,339,172 | *NRIP1* | G | C | 0.002 | 0.007 |
| 21 | rs75518158 | 38,393,567 | *DSCR6* | A | C | 0.015 | 0.013 |
| 21 | rs220193 | 43,581,308 | *UMODL1* | G | A | 0.001 | 0.006 |
| 22 | rs134594 | 29,468,456 | *KREMEN1* | T | C | 0.009 | 0.005 |
| 22 | rs5750561 | 38,595,260 | *MAFF* | A | T | 0.006 | 0.005 |
| 22 | rs41311445 | 42,070,374 | *NHP2L1/SREBF2* | C | A | 0.002 | 0.008 |
| 22 | rs11704481 | 45,732,328 | *FAM118A* | G | A | 0.002 | 0.005 |
| 22 | rs7285579 | 46,441,980 | *LOC100271722* | T | C | 0.002 | 0.005 |
| 22 | rs28681372 | 50,351,977 | *PIM3* | G | A | 0.003 | 0.005 |
| 22 | rs28681372 | 50,351,977 | *PIM3* | G | A | 0.003 | 0.005 |

Table S4. Genetic correlation between the fetal/maternal specific effect of birth weight and stroke as well as its subtypes

| stroke | *r_g_* | *se* | Z | *p* value |
| --- | --- | --- | --- | --- |
| fetal effect of birth weight | | | | |
| AIS | 0.007 | 0.046 | 0.144 | 0.886 |
| CES | 0.036 | 0.072 | 0.496 | 0.620 |
| LAS | NA | | | |
| SVS | NA | | | |
| maternal effect of birth weight | | | | |
| AIS | -0.176 | 0.054 | -3.272 | 0.001 |
| CES | -0.005 | 0.082 | -0.056 | 0.955 |
| LAS | NA | | | |
| SVS | NA | | | |

Note: In our LDSC analysis, only maternal genetic foundation between birth weight and stroke was found while no fetal genetic correlation between birth weight and stroke was detected. However, a negative heritability was calculated on the stroke subtypes (LAS and SVS), which made it impossible to estimate genetic correlation. The negative heritability may due to the low true heritability and the sampling error (especially if the sample size is small and thus there’s lots of sampling variation) ([Bulik-Sullivan et al., 2015](#_ENREF_6)).

Table S5. Traits and diseases that were associated with instrumental variables of birth weight

| SNP | Chr | Position | Traits or diseases |
| --- | --- | --- | --- |
| rs17367504 | 1 | 11,862,778 | blood pressure, diastolic blood pressure, mean arterial pressure, pulse pressure, systolic blood pressure |
| rs905938 | 1 | 154,991,389 | birth length, waist-hip ratio |
| rs11893688 | 2 | 9,695,282 | waist-hip ratio |
| rs560887 | 2 | 169,763,148 | fasting blood glucose, fasting plasma glucose, glycated hemoglobin levels, glycemic traits, hemoglobin A1c levels, metabolic syndrome, metabolic traits, pulse pressure |
| rs11708067 | 3 | 123,065,778 | fasting blood glucose, glycated hemoglobin levels, glycemic traits, type 2 diabetes |
| rs1482852 | 3 | 156,798,294 | waist circumference adjusted for BMI (joint analysis main effects and physical activity interaction), waist-to-hip ratio adjusted for BMI |
| rs11545169 | 3 | 184,020,542 | educational attainment, height |
| rs6845999 | 4 | 145,565,826 | height |
| rs2131354 | 4 | 145,599,908 | height, hip circumference adjusted for BMI |
| rs12656216 | 5 | 36,160,668 | height |
| rs1818782 | 5 | 39,424,628 | height |
| rs6871635 | 5 | 133,830,395 | educational attainment (years of education) |
| rs12153596 | 5 | 158,410,178 | height |
| rs34471628 | 5 | 172,196,752 | c-reactive protein levels |
| rs9379084 | 6 | 7,231,843 | height, type 2 diabetes |
| rs35261542 | 6 | 20,675,792 | body mass index, hemoglobin A1c levels, type 2 diabetes |
| rs7744700 | 6 | 53,349,401 | height |
| rs1415701 | 6 | 130,345,835 | height |
| rs34776209 | 7 | 23,513,093 | fat-free mass |
| rs2908279 | 7 | 44,174,857 | type 2 diabetes |
| rs2282978 | 7 | 92,264,410 | diastolic blood pressure, height, pulse pressure, systolic blood pressure |
| rs45446698 | 7 | 99,332,948 | blood protein levels, height |
| rs3918226 | 7 | 150,690,176 | cardiovascular disease, coronary artery disease, diastolic blood pressure, height, systolic blood pressure |
| rs72656010 | 8 | 57,122,215 | fat-free mass, height |
| rs10283100 | 8 | 120,596,023 | height |
| rs10814916 | 9 | 4,293,150 | type 2 diabetes |
| rs2274224 | 10 | 96,039,597 | body fat percentage |
| rs7903146 | 10 | 114,758,349 | body mass index, fasting blood glucose, fasting blood insulin, glucosuria, glycated hemoglobin levels, hip circumference, metabolic syndrome, peak insulin response, peripheral artery disease, proinsulin levels, pulse pressure, systolic blood pressure, type 2 diabetes, waist circumference |
| rs7076938 | 10 | 115,789,375 | mean arterial pressure |
| rs1801253 | 10 | 115,805,056 | cardiovascular disease, diastolic blood pressure, height, systolic blood pressure |
| rs2292626 | 10 | 124,186,714 | type 2 diabetes |
| rs2168101 | 11 | 8,255,408 | height |
| rs10830963 | 11 | 92,708,710 | acute insulin response, corrected insulin response, fasting blood glucose, fasting plasma glucose, glucose homeostasis traits, glycated hemoglobin levels, glycemic traits, hemoglobin A1c levels, incremental insulin, insulin disposition index, insulin levels, metabolite levels, obesity-related traits, peak insulin response, pulse pressure, type 2 diabetes |
| rs76895963 | 12 | 4,384,844 | body mass index, cardiovascular disease, height, pulse pressure, systolic blood pressure, type 2 diabetes |
| rs8756 | 12 | 66,359,752 | height |
| rs7968682 | 12 | 66,371,880 | height |
| rs3184504 | 12 | 111,884,608 | blood metabolite levels, blood protein levels, body mass index, cancer, cardiovascular disease, coronary heart disease, diastolic blood pressure, fibrinogen levels, granulocyte count, height, hemoglobin concentration, high density lipoprotein cholesterol levels, hypothyroidism, IGA levels, low density lipoprotein cholesterol levels, systolic blood pressure, total cholesterol levels, type 1 diabetes, white blood cell count |
| rs72681869 | 14 | 50,655,357 | body mass index, cardiovascular disease, intraocular pressure, systolic blood pressure |
| rs5742915 | 15 | 74,336,633 | fat-free mass, height |
| rs12909648 | 15 | 86,224,570 | systolic blood pressure |
| rs4932373 | 15 | 91,429,287 | coronary artery disease |
| rs2428362 | 17 | 7,180,274 | systolic blood pressure |
| rs78378222 | 17 | 7,571,752 | diastolic blood pressure, fat-free mass, mean corpuscular hemoglobin, pulse pressure, red blood cell count, white blood cell count |
| rs7223535 | 17 | 29,211,667 | fat-free mass, height |
| rs11867479 | 17 | 68,090,207 | height |
| rs11082304 | 18 | 20,720,973 | height, platelet count, smoking behavior |
| rs516246 | 19 | 49,206,172 | blood protein levels, cardiovascular disease, low density lipoprotein cholesterol levels, obesity-related traits, total cholesterol levels, triglycerides, type 1 diabetes |
| rs147110934 | 19 | 55,993,436 | fat-free mass, height |
| rs73143584 | 20 | 62,445,702 | coronary artery disease |
| rs2229742 | 21 | 16,339,172 | red blood cell count, systolic blood pressure |

We searched the GWAS catalog (<https://www.ebi.ac.uk/gwas>; until 10/29/2019) to check if there existed instrumental variables which showed any associations with other traits or diseases.

**MEGASTROKE Consortium**

Rainer Malik 1, Ganesh Chauhan 2, Matthew Traylor 3, Muralidharan Sargurupremraj 4,5, Yukinori Okada 6,7,8, Aniket Mishra 4,5, Loes Rutten-Jacobs 3, Anne-Katrin Giese 9, Sander W van der Laan 10, Solveig Gretarsdottir 11, Christopher D Anderson 12,13,14,14, Michael Chong 15, Hieab HH Adams 16,17, Tetsuro Ago 18, Peter Almgren 19, Philippe Amouyel 20,21, Hakan Ay 22,13, Traci M Bartz 23, Oscar R Benavente 24, Steve Bevan 25, Giorgio B Boncoraglio 26, Robert D Brown, Jr. 27, Adam S Butterworth 28,29, Caty Carrera 30,31, Cara L Carty 32,33, Daniel I Chasman 34,35, Wei-Min Chen 36, John W Cole 37, Adolfo Correa 38, Ioana Cotlarciuc 39, Carlos Cruchaga 40,41, John Danesh 28,42,43,44, Paul IW de Bakker 45,46, Anita L DeStefano 47,48, Marcel den Hoed 49, Qing Duan 50, Stefan T Engelter 51,52, Guido J Falcone 53,54, Rebecca F Gottesman 55, Raji P Grewal 56, Vilmundur Gudnason 57,58, Stefan Gustafsson 59, Jeffrey Haessler 60, Tamara B Harris 61, Ahamad Hassan 62, Aki S Havulinna 63,64, Susan R Heckbert 65, Elizabeth G Holliday 66,67, George Howard 68, Fang-Chi Hsu 69, Hyacinth I Hyacinth 70, M Arfan Ikram 16, Erik Ingelsson 71,72, Marguerite R Irvin 73, Xueqiu Jian 74, Jordi Jiménez-Conde 75, Julie A Johnson 76,77, J Wouter Jukema 78, Masahiro Kanai 6,7,79, Keith L Keene 80,81, Brett M Kissela 82, Dawn O Kleindorfer 82, Charles Kooperberg 60, Michiaki Kubo 83, Leslie A Lange 84, Carl D Langefeld 85, Claudia Langenberg 86, Lenore J Launer 87, Jin-Moo Lee 88, Robin Lemmens 89,90, Didier Leys 91, Cathryn M Lewis 92,93, Wei-Yu Lin 28,94, Arne G Lindgren 95,96, Erik Lorentzen 97, Patrik K Magnusson 98, Jane Maguire 99, Ani Manichaikul 36, Patrick F McArdle 100, James F Meschia 101, Braxton D Mitchell 100,102, Thomas H Mosley 103,104, Michael A Nalls 105,106, Toshiharu Ninomiya 107, Martin J O'Donnell 15,108, Bruce M Psaty 109,110,111,112, Sara L Pulit 113,45, Kristiina Rannikmäe 114,115, Alexander P Reiner 65,116, Kathryn M Rexrode 117, Kenneth Rice 118, Stephen S Rich 36, Paul M Ridker 34,35, Natalia S Rost 9,13, Peter M Rothwell 119, Jerome I Rotter 120,121, Tatjana Rundek 122, Ralph L Sacco 122, Saori Sakaue 7,123, Michele M Sale 124, Veikko Salomaa 63, Bishwa R Sapkota 125, Reinhold Schmidt 126, Carsten O Schmidt 127, Ulf Schminke 128, Pankaj Sharma 39, Agnieszka Slowik 129, Cathie LM Sudlow 114,115, Christian Tanislav 130, Turgut Tatlisumak 131,132, Kent D Taylor 120,121, Vincent NS Thijs 133,134, Gudmar Thorleifsson 11, Unnur Thorsteinsdottir 11, Steffen Tiedt 1, Stella Trompet 135, Christophe Tzourio 5,136,137, Cornelia M van Duijn 138,139, Matthew Walters 140, Nicholas J Wareham 86, Sylvia Wassertheil-Smoller 141, James G Wilson 142, Kerri L Wiggins 109, Qiong Yang 47, Salim Yusuf 15, Najaf Amin 16, Hugo S Aparicio 185,48, Donna K Arnett 186, John Attia 187, Alexa S Beiser 47,48, Claudine Berr 188, Julie E Buring 34,35, Mariana Bustamante 189, Valeria Caso 190, Yu-Ching Cheng 191, Seung Hoan Choi 192,48, Ayesha Chowhan 185,48, Natalia Cullell 31, Jean-François Dartigues 193,194, Hossein Delavaran 95,96, Pilar Delgado 195, Marcus Dörr 196,197, Gunnar Engström 19, Ian Ford 198, Wander S Gurpreet 199, Anders Hamsten 200,201, Laura Heitsch 202, Atsushi Hozawa 203, Laura Ibanez 204, Andreea Ilinca 95,96, Martin Ingelsson 205, Motoki Iwasaki 206, Rebecca D Jackson 207, Katarina Jood 208, Pekka Jousilahti 63, Sara Kaffashian 4,5, Lalit Kalra 209, Masahiro Kamouchi 210, Takanari Kitazono 211, Olafur Kjartansson 212, Manja Kloss 213, Peter J Koudstaal 214, Jerzy Krupinski 215, Daniel L Labovitz 216, Cathy C Laurie 118, Christopher R Levi 217, Linxin Li 218, Lars Lind 219, Cecilia M Lindgren 220,221, Vasileios Lioutas 222,48, Yong Mei Liu 223, Oscar L Lopez 224, Hirata Makoto 225, Nicolas Martinez-Majander 172, Koichi Matsuda 225, Naoko Minegishi 203, Joan Montaner 226, Andrew P Morris 227,228, Elena Muiño 31, Martina Müller-Nurasyid 229,230,231, Bo Norrving 95,96, Soichi Ogishima 203, Eugenio A Parati 232, Leema Reddy Peddareddygari 56, Nancy L Pedersen 98,233, Joanna Pera 129, Markus Perola 63,234, Alessandro Pezzini 235, Silvana Pileggi 236, Raquel Rabionet 237, Iolanda Riba-Llena 30, Marta Ribasés 238, Jose R Romero 185,48, Jaume Roquer 239,240, Anthony G Rudd 241,242, Antti-Pekka Sarin 243,244, Ralhan Sarju 199, Chloe Sarnowski 47,48, Makoto Sasaki 245, Claudia L Satizabal 185,48, Mamoru Satoh 245, Naveed Sattar 246, Norie Sawada 206, Gerli Sibolt 172, Ásgeir Sigurdsson 247, Albert Smith 248, Kenji Sobue 245, Carolina Soriano-Tárraga 240, Tara Stanne 249, O Colin Stine 250, David J Stott 251, Konstantin Strauch 229,252, Takako Takai 203, Hideo Tanaka 253,254, Kozo Tanno 245, Alexander Teumer 255, Liisa Tomppo 172, Nuria P Torres-Aguila 31, Emmanuel Touze 256,257, Shoichiro Tsugane 206, Andre G Uitterlinden 258, Einar M Valdimarsson 259, Sven J van der Lee 16, Henry Völzke 255, Kenji Wakai 253, David Weir 260, Stephen R Williams 261, Charles DA Wolfe 241,242, Quenna Wong 118, Huichun Xu 191, Taiki Yamaji 206, Dharambir K Sanghera 125,169,170, Olle Melander 19, Christina Jern 171, Daniel Strbian 172,173, Israel Fernandez-Cadenas 31,30, W T Longstreth, Jr 174,65, Arndt Rolfs 175, Jun Hata 107, Daniel Woo 82, Jonathan Rosand 12,13,14, Guillaume Pare 15, Jemma C Hopewell 176, Danish Saleheen 177, Kari Stefansson 11,178, Bradford B Worrall 179, Steven J Kittner 37, Sudha Seshadri 180,48, Myriam Fornage 74,181, Hugh S Markus 3, Joanna MM Howson 28, Yoichiro Kamatani 6,182, Stephanie Debette 4,5, Martin Dichgans 1,183,184

1 Institute for Stroke and Dementia Research (ISD), University Hospital, LMU Munich, Munich, Germany

2 Centre for Brain Research, Indian Institute of Science, Bangalore, India

3 Stroke Research Group, Division of Clinical Neurosciences, University of Cambridge, UK

4 INSERM U1219 Bordeaux Population Health Research Center, Bordeaux, France

5 University of Bordeaux, Bordeaux, France

6 Laboratory for Statistical Analysis, RIKEN Center for Integrative Medical Sciences, Yokohama, Japan

7 Department of Statistical Genetics, Osaka University Graduate School of Medicine, Osaka, Japan

8 Laboratory of Statistical Immunology, Immunology Frontier Research Center (WPI-IFReC), Osaka University, Suita, Japan.

9 Department of Neurology, Massachusetts General Hospital, Harvard Medical School, Boston, MA, USA

10 Laboratory of Experimental Cardiology, Division of Heart and Lungs, University Medical Center Utrecht, University of Utrecht, Utrecht,Netherlands

11 deCODE genetics/AMGEN inc, Reykjavik, Iceland

12 Center for Genomic Medicine, Massachusetts General Hospital (MGH), Boston, MA, USA

13 J. Philip Kistler Stroke Research Center, Department of Neurology, MGH, Boston, MA, USA

14 Program in Medical and Population Genetics, Broad Institute, Cambridge, MA, USA

15 Population Health Research Institute, McMaster University, Hamilton, Canada

16 Department of Epidemiology, Erasmus University Medical Center, Rotterdam, Netherlands

17 Department of Radiology and Nuclear Medicine, Erasmus University Medical Center, Rotterdam, Netherlands

18 Department of Medicine and Clinical Science, Graduate School of Medical Sciences, Kyushu University, Fukuoka, Japan

19 Department of Clinical Sciences, Lund University, Malmö, Sweden

20 Univ. Lille, Inserm, Institut Pasteur de Lille, LabEx DISTALZ-UMR1167, Risk factors and molecular determinants of aging-related diseases, F-59000 Lille, France

21 Centre Hosp. Univ Lille, Epidemiology and Public Health Department, F-59000 Lille, France

22 AA Martinos Center for Biomedical Imaging, Department of Radiology, Massachusetts General Hospital, Harvard Medical School, Boston, MA, USA

23 Cardiovascular Health Research Unit, Departments of Biostatistics and Medicine, University of Washington, Seattle, WA, USA

24 Division of Neurology, Faculty of Medicine, Brain Research Center, University of British Columbia, Vancouver, Canada

25 School of Life Science, University of Lincoln, Lincoln, UK

26 Department of Cerebrovascular Diseases, Fondazione IRCCS Istituto Neurologico "Carlo Besta", Milano, Italy

27 Department of Neurology, Mayo Clinic Rochester, Rochester, MN, USA

28 MRC/BHF Cardiovascular Epidemiology Unit, Department of Public Health and Primary Care, University of Cambridge, Cambridge, UK

29 The National Institute for Health Research Blood and Transplant Research Unit in Donor Health and Genomics, University of Cambridge, UK

30 Neurovascular Research Laboratory, Vall d'Hebron Institut of Research, Neurology and Medicine Departments-Universitat Autònoma de Barcelona, Vall d’Hebrón Hospital, Barcelona, Spain

31 Stroke Pharmacogenomics and Genetics, Fundacio Docència i Recerca MutuaTerrassa, Terrassa, Spain

32 Children's Research Institute, Children's National Medical Center, Washington, DC, USA

33 Center for Translational Science, George Washington University, Washington, DC, USA

34 Division of Preventive Medicine, Brigham and Women's Hospital, Boston, MA, USA

35 Harvard Medical School, Boston, MA, USA

36 Center for Public Health Genomics, Department of Public Health Sciences, University of Virginia, Charlottesville, VA, USA

37 Department of Neurology, University of Maryland School of Medicine and Baltimore VAMC, Baltimore, MD, USA

38 Departments of Medicine, Pediatrics and Population Health Science, University of Mississippi Medical Center, Jackson, MS, USA

39 Institute of Cardiovascular Research, Royal Holloway University of London, UK & Ashford and St Peters Hospital, Surrey UK

40 Department of Psychiatry,The Hope Center Program on Protein Aggregation and Neurodegeneration (HPAN),Washington University, School of Medicine, St. Louis, MO, USA

41 Department of Developmental Biology, Washington University School of Medicine, St. Louis, MO, USA

42 NIHR Blood and Transplant Research Unit in Donor Health and Genomics, Department of Public Health and Primary Care, University of Cambridge, Cambridge, UK

43 Wellcome Trust Sanger Institute, Wellcome Trust Genome Campus, Hinxton, Cambridge, UK

44 British Heart Foundation, Cambridge Centre of Excellence, Department of Medicine, University of Cambridge, Cambridge, UK

45 Department of Medical Genetics, University Medical Center Utrecht, Utrecht, Netherlands

46 Department of Epidemiology, Julius Center for Health Sciences and Primary Care, University Medical Center Utrecht, Utrecht, Netherlands

47 Boston University School of Public Health, Boston, MA, USA

48 Framingham Heart Study, Framingham, MA, USA

49 Department of Immunology, Genetics and Pathology and Science for Life Laboratory, Uppsala University, Uppsala, Sweden

50 Department of Genetics, University of North Carolina, Chapel Hill, NC, USA

51 Department of Neurology and Stroke Center, Basel University Hospital, Switzerland

52 Neurorehabilitation Unit, University and University Center for Medicine of Aging and Rehabilitation Basel, Felix Platter Hospital, Basel, Switzerland

53 Department of Neurology, Yale University School of Medicine, New Haven, CT, USA

54 Program in Medical and Population Genetics, The Broad Institute of Harvard and MIT, Cambridge, MA, USA

55 Department of Neurology, Johns Hopkins University School of Medicine, Baltimore, MD, USA

56 Neuroscience Institute, SF Medical Center, Trenton, NJ, USA

57 Icelandic Heart Association Research Institute, Kopavogur, Iceland

58 University of Iceland, Faculty of Medicine, Reykjavik, Iceland

59 Department of Medical Sciences, Molecular Epidemiology and Science for Life Laboratory, Uppsala University, Uppsala, Sweden

60 Division of Public Health Sciences, Fred Hutchinson Cancer Research Center, Seattle, WA, USA

61 Laboratory of Epidemiology and Population Science, National Institute on Aging, National Institutes of Health, Bethesda, MD, USA

62 Department of Neurology, Leeds General Infirmary, Leeds Teaching Hospitals NHS Trust, Leeds, UK

63 National Institute for Health and Welfare, Helsinki, Finland

64 FIMM - Institute for Molecular Medicine Finland, Helsinki, Finland

65 Department of Epidemiology, University of Washington, Seattle, WA, USA

66 Public Health Stream, Hunter Medical Research Institute, New Lambton, Australia

67 Faculty of Health and Medicine, University of Newcastle, Newcastle, Australia

68 School of Public Health, University of Alabama at Birmingham, Birmingham, AL, USA

69 Department of Biostatistical Sciences, Wake Forest School of Medicine, Winston-Salem, NC, USA

70 Aflac Cancer and Blood Disorder Center, Department of Pediatrics, Emory University School of Medicine, Atlanta, GA, USA

71 Department of Medicine, Division of Cardiovascular Medicine, Stanford University School of Medicine, CA, USA

72 Department of Medical Sciences, Molecular Epidemiology and Science for Life Laboratory, Uppsala University, Uppsala, Sweden

73 Epidemiology, School of Public Health, University of Alabama at Birmingham, USA

74 Brown Foundation Institute of Molecular Medicine, University of Texas Health Science Center at Houston, Houston, TX, USA

75 Neurovascular Research Group (NEUVAS), Neurology Department, Institut Hospital del Mar d'Investigació Mèdica, Universitat Autònoma de Barcelona, Barcelona, Spain

76 Department of Pharmacotherapy and Translational Research and Center for Pharmacogenomics, University of Florida, College of Pharmacy, Gainesville, FL, USA

77 Division of Cardiovascular Medicine, College of Medicine, University of Florida, Gainesville, FL, USA

78 Department of Cardiology, Leiden University Medical Center, Leiden, the Netherlands

79 Program in Bioinformatics and Integrative Genomics, Harvard Medical School, Boston, MA, USA

80 Department of Biology, East Carolina University, Greenville, NC, USA

81 Center for Health Disparities, East Carolina University, Greenville, NC, USA

82 University of Cincinnati College of Medicine, Cincinnati, OH, USA

83 RIKEN Center for Integrative Medical Sciences, Yokohama, Japan

84 Department of Medicine, University of Colorado Denver, Anschutz Medical Campus, Aurora, CO, USA

85 Center for Public Health Genomics and Department of Biostatistical Sciences, Wake Forest School of Medicine, Winston-Salem, NC, USA

86 MRC Epidemiology Unit, University of Cambridge School of Clinical Medicine, Institute of Metabolic Science, Cambridge Biomedical Campus, Cambridge, UK

87 Intramural Research Program, National Institute on Aging, National Institutes of Health, Bethesda, MD, USA

88 Department of Neurology, Radiology, and Biomedical Engineering, Washington University School of Medicine, St. Louis, MO, USA

89 KU Leuven – University of Leuven, Department of Neurosciences, Experimental Neurology, Leuven, Belgium

90 VIB Center for Brain & Disease Research, University Hospitals Leuven, Department of Neurology, Leuven, Belgium

91 Univ.-Lille, INSERM U 1171. CHU Lille. Lille, France

92 Department of Medical and Molecular Genetics, King's College London, London, UK

93 SGDP Centre, Institute of Psychiatry, Psychology & Neuroscience, King's College London, London, UK

94 Northern Institute for Cancer Research, Paul O'Gorman Building, Newcastle University, Newcastle, UK

95 Department of Clinical Sciences Lund, Neurology, Lund University, Lund, Sweden

96 Department of Neurology and Rehabilitation Medicine, Skåne University Hospital, Lund, Sweden

97 Bioinformatics Core Facility, University of Gothenburg, Gothenburg, Sweden

98 Department of Medical Epidemiology and Biostatistics, Karolinska Institutet, Stockholm, Sweden

99 University of Technology Sydney, Faculty of Health, Ultimo, Australia

100 Department of Medicine, University of Maryland School of Medicine, MD, USA

101 Department of Neurology, Mayo Clinic, Jacksonville, FL, USA

102 Geriatrics Research and Education Clinical Center, Baltimore Veterans Administration Medical Center, Baltimore, MD, USA

103 Division of Geriatrics, School of Medicine, University of Mississippi Medical Center, Jackson, MS, USA

104 Memory Impairment and Neurodegenerative Dementia Center, University of Mississippi Medical Center, Jackson, MS, USA

105 Laboratory of Neurogenetics, National Institute on Aging, National institutes of Health, Bethesda, MD, USA

106 Data Tecnica International, Glen Echo MD, USA

107 Department of Epidemiology and Public Health, Graduate School of Medical Sciences, Kyushu University, Fukuoka, Japan

108 Clinical Research Facility, Department of Medicine, NUI Galway, Galway, Ireland

109 Cardiovascular Health Research Unit, Department of Medicine, University of Washington, Seattle, WA, USA

110 Department of Epidemiology, University of Washington, Seattle, WA

111 Department of Health Services, University of Washington, Seattle, WA, USA

112 Kaiser Permanente Washington Health Research Institute, Seattle, WA, USA

113 Brain Center Rudolf Magnus, Department of Neurology, University Medical Center Utrecht, Utrecht, The Netherlands

114 Usher Institute of Population Health Sciences and Informatics, University of Edinburgh, Edinburgh, UK

115 Centre for Clinical Brain Sciences, University of Edinburgh, Edinburgh, UK

116 Fred Hutchinson Cancer Research Center, University of Washington, Seattle, WA, USA

117 Department of Medicine, Brigham and Women's Hospital, Boston, MA, USA

118 Department of Biostatistics, University of Washington, Seattle, WA, USA

119 Nuffield Department of Clinical Neurosciences, University of Oxford, UK

120 Institute for Translational Genomics and Population Sciences, Los Angeles Biomedical Research Institute at Harbor-UCLA Medical Center, Torrance, CA, USA

121 Division of Genomic Outcomes, Department of Pediatrics, Harbor-UCLA Medical Center, Torrance, CA, USA

122 Department of Neurology, Miller School of Medicine, University of Miami, Miami, FL, USA

123 Department of Allergy and Rheumatology, Graduate School of Medicine, the University of Tokyo, Tokyo, Japan

124 Center for Public Health Genomics, University of Virginia, Charlottesville, VA, USA

125 Department of Pediatrics, College of Medicine, University of Oklahoma Health Sciences Center, Oklahoma City, OK, USA

126 Department of Neurology, Medical University of Graz, Graz, Austria

127 University Medicine Greifswald, Institute for Community Medicine, SHIP-KEF, Greifswald, Germany

128 University Medicine Greifswald, Department of Neurology, Greifswald, Germany

129 Department of Neurology, Jagiellonian University, Krakow, Poland

130 Department of Neurology, Justus Liebig University, Giessen, Germany

131 Department of Clinical Neurosciences/Neurology, Institute of Neuroscience and Physiology, Sahlgrenska Academy at University of Gothenburg, Gothenburg, Sweden

132 Sahlgrenska University Hospital, Gothenburg, Sweden

133 Stroke Division, Florey Institute of Neuroscience and Mental Health, University of Melbourne, Heidelberg, Australia

134 Austin Health, Department of Neurology, Heidelberg, Australia

135 Department of Internal Medicine, Section Gerontology and Geriatrics, Leiden University Medical Center, Leiden, the Netherlands

136 INSERM U1219, Bordeaux, France

137 Department of Public Health, Bordeaux University Hospital, Bordeaux, France

138 Genetic Epidemiology Unit, Department of Epidemiology, Erasmus University Medical Center Rotterdam, Netherlands

139 Center for Medical Systems Biology, Leiden, Netherlands

140 School of Medicine, Dentistry and Nursing at the University of Glasgow, Glasgow, UK

141 Department of Epidemiology and Population Health, Albert Einstein College of Medicine, NY, USA

142 Department of Physiology and Biophysics, University of Mississippi Medical Center, Jackson, MS, USA

143 A full list of members and affiliations appears in the Supplementary Note

144 Department of Human Genetics, McGill University, Montreal, Canada

145 Department of Pathophysiology, Institute of Biomedicine and Translation Medicine, University of Tartu, Tartu, Estonia

146 Department of Cardiac Surgery, Tartu University Hospital, Tartu, Estonia

147 Clinical Gene Networks AB,Stockholm, Sweden

148 Department of Genetics and Genomic Sciences, The Icahn Institute for Genomics and Multiscale Biology Icahn School of Medicine at Mount Sinai, New York, NY, USA

149 Department of Pathophysiology, Institute of Biomedicine and Translation Medicine, University of Tartu, Biomeedikum, Tartu, Estonia

150 Integrated Cardio Metabolic Centre, Department of Medicine, Karolinska Institutet, Karolinska Universitetssjukhuset, Huddinge, Sweden.

151 Clinical Gene Networks AB, Stockholm, Sweden

152 Sorbonne Universités, UPMC Univ. Paris 06, INSERM, UMR_S 1166, Team Genomics & Pathophysiology of Cardiovascular Diseases, Paris, France

153 ICAN Institute for Cardiometabolism and Nutrition, Paris, France

154 Department of Biomedical Engineering, University of Virginia, Charlottesville, VA, USA

155 Group Health Research Institute, Group Health Cooperative, Seattle, WA, USA

156 Seattle Epidemiologic Research and Information Center, VA Office of Research and Development, Seattle, WA, USA

157 Cardiovascular Research Center, Massachusetts General Hospital, Boston, MA, USA

158 Department of Medical Research, Bærum Hospital, Vestre Viken Hospital Trust, Gjettum, Norway

159 Saw Swee Hock School of Public Health, National University of Singapore and National University Health System, Singapore

160 National Heart and Lung Institute, Imperial College London, London, UK

161 Department of Gene Diagnostics and Therapeutics, Research Institute, National Center for Global Health and Medicine, Tokyo, Japan

162 Department of Epidemiology, Tulane University School of Public Health and Tropical Medicine, New Orleans, LA, USA

163 Department of Cardiology,University Medical Center Groningen, University of Groningen, Netherlands

164 MRC-PHE Centre for Environment and Health, School of Public Health, Department of Epidemiology and Biostatistics, Imperial College London, London, UK

165 Department of Epidemiology and Biostatistics, Imperial College London, London, UK

166 Department of Cardiology, Ealing Hospital NHS Trust, Southall, UK

167 National Heart, Lung and Blood Research Institute, Division of Intramural Research, Population Sciences Branch, Framingham, MA, USA

168 A full list of members and affiliations appears at the end of the manuscript

169 Department of Phamaceutical Sciences, Collge of Pharmacy, University of Oklahoma Health Sciences Center, Oklahoma City, OK, USA

170 Oklahoma Center for Neuroscience, Oklahoma City, OK, USA

171 Department of Pathology and Genetics, Institute of Biomedicine, The Sahlgrenska Academy at University of Gothenburg, Gothenburg, Sweden

172 Department of Neurology, Helsinki University Hospital, Helsinki, Finland

173 Clinical Neurosciences, Neurology, University of Helsinki, Helsinki, Finland

174 Department of Neurology, University of Washington, Seattle, WA, USA

175 Albrecht Kossel Institute, University Clinic of Rostock, Rostock, Germany

176 Clinical Trial Service Unit and Epidemiological Studies Unit, Nuffield Department of Population Health, University of Oxford, Oxford, UK

177 Department of Genetics, Perelman School of Medicine, University of Pennsylvania, PA, USA

178 Faculty of Medicine, University of Iceland, Reykjavik, Iceland

179 Departments of Neurology and Public Health Sciences, University of Virginia School of Medicine, Charlottesville, VA, USA

180 Department of Neurology, Boston University School of Medicine, Boston, MA, USA

181 Human Genetics Center, University of Texas Health Science Center at Houston, Houston, TX, USA

182 Center for Genomic Medicine, Kyoto University Graduate School of Medicine, Kyoto, Japan

183 Munich Cluster for Systems Neurology (SyNergy), Munich, Germany

184 German Center for Neurodegenerative Diseases (DZNE), Munich, Germany

185 Boston University School of Medicine, Boston, MA, USA

186 University of Kentucky College of Public Health, Lexington, KY, USA

187 University of Newcastle and Hunter Medical Research Institute, New Lambton, Australia

188 Univ. Montpellier, Inserm, U1061, Montpellier, France

189 Centre for Research in Environmental Epidemiology, Barcelona, Spain

190 Department of Neurology, Università degli Studi di Perugia, Umbria, Italy

191 Department of Medicine, University of Maryland School of Medicine, Baltimore, MD, USA

192 Broad Institute, Cambridge, MA, USA

193 Univ. Bordeaux, Inserm, Bordeaux Population Health Research Center, UMR 1219, Bordeaux, France

194 Bordeaux University Hospital, Department of Neurology, Memory Clinic, Bordeaux, France

195 Neurovascular Research Laboratory. Vall d'Hebron Institut of Research, Neurology and Medicine Departments-Universitat Autònoma de Barcelona. Vall d’Hebrón Hospital, Barcelona, Spain

196 University Medicine Greifswald, Department of Internal Medicine B, Greifswald, Germany

197 DZHK, Greifswald, Germany

198 Robertson Center for Biostatistics, University of Glasgow, Glasgow, UK

199 Hero DMC Heart Institute, Dayanand Medical College & Hospital, Ludhiana, India

200 Atherosclerosis Research Unit, Department of Medicine Solna, Karolinska Institutet, Stockholm, Sweden

201 Karolinska Institutet, Stockholm, Sweden

202 Division of Emergency Medicine, and Department of Neurology, Washington University School of Medicine, St. Louis, MO, USA

203 Tohoku Medical Megabank Organization, Sendai, Japan

204 Department of Psychiatry, Washington University School of Medicine, St. Louis, MO, USA

205 Department of Public Health and Caring Sciences / Geriatrics, Uppsala University, Uppsala, Sweden

206 Epidemiology and Prevention Group, Center for Public Health Sciences, National Cancer Center, Tokyo, Japan

207 Department of Internal Medicine and the Center for Clinical and Translational Science, The Ohio State University, Columbus, OH, USA

208 Institute of Neuroscience and Physiology, the Sahlgrenska Academy at University of Gothenburg, Goteborg, Sweden

209 Department of Basic and Clinical Neurosciences, King's College London, London, UK

210 Department of Health Care Administration and Management, Graduate School of Medical Sciences, Kyushu University, Japan

211 Department of Medicine and Clinical Science, Graduate School of Medical Sciences, Kyushu University, Japan

212 Landspitali National University Hospital, Departments of Neurology & Radiology, Reykjavik, Iceland

213 Department of Neurology, Heidelberg University Hospital, Germany

214 Department of Neurology, Erasmus University Medical Center

215 Hospital Universitari Mutua Terrassa, Terrassa (Barcelona), Spain

216 Albert Einstein College of Medicine, Montefiore Medical Center, New York, NY, USA

217 John Hunter Hospital, Hunter Medical Research Institute and University of Newcastle, Newcastle, NSW, Australia

218 Centre for Prevention of Stroke and Dementia, Nuffield Department of Clinical Neurosciences, University of Oxford, UK

219 Department of Medical Sciences, Uppsala University, Uppsala, Sweden

220 Genetic and Genomic Epidemiology Unit, Wellcome Trust Centre for Human Genetics, University of Oxford, Oxford, UK

221 The Wellcome Trust Centre for Human Genetics, Oxford, UK

222 Beth Israel Deaconess Medical Center, Boston, MA, USA

223 Wake Forest School of Medicine, Wake Forest, NC, USA

224 Department of Neurology, University of Pittsburgh, Pittsburgh, PA, USA

225 BioBank Japan, Laboratory of Clinical Sequencing, Department of Computational biology and medical Sciences, Graduate school of Frontier Sciences, The University of Tokyo, Tokyo, Japan

226 Neurovascular Research Laboratory, Vall d'Hebron Institut of Research, Neurology and Medicine Departments-Universitat Autònoma de Barcelona. Vall d’Hebrón Hospital, Barcelona, Spain

227 Department of Biostatistics, University of Liverpool, Liverpool, UK

228 Wellcome Trust Centre for Human Genetics, University of Oxford, Oxford, UK

229 Institute of Genetic Epidemiology, Helmholtz Zentrum München - German Research Center for Environmental Health, Neuherberg, Germany

230 Department of Medicine I, Ludwig-Maximilians-Universität, Munich, Germany

231 DZHK (German Centre for Cardiovascular Research), partner site Munich Heart Alliance, Munich, Germany

232 Department of Cerebrovascular Diseases, Fondazione IRCCS Istituto Neurologico “Carlo Besta”, Milano, Italy

233 Karolinska Institutet, MEB, Stockholm, Sweden

234 University of Tartu, Estonian Genome Center, Tartu, Estonia, Tartu, Estonia

235 Department of Clinical and Experimental Sciences, Neurology Clinic, University of Brescia, Italy

236 Translational Genomics Unit, Department of Oncology, IRCCS Istituto di Ricerche Farmacologiche Mario Negri, Milano, Italy

237 Department of Genetics, Microbiology and Statistics, University of Barcelona, Barcelona, Spain

238 Psychiatric Genetics Unit, Group of Psychiatry, Mental Health and Addictions, Vall d’Hebron Research Institute (VHIR), Universitat Autònoma de Barcelona, Biomedical Network Research Centre on Mental Health (CIBERSAM), Barcelona, Spain

239 Department of Neurology, IMIM-Hospital del Mar, and Universitat Autònoma de Barcelona, Spain

240 IMIM (Hospital del Mar Medical Research Institute), Barcelona, Spain

241 National Institute for Health Research Comprehensive Biomedical Research Centre, Guy's & St. Thomas' NHS Foundation Trust and King's College London, London, UK

242 Division of Health and Social Care Research, King's College London, London, UK

243 FIMM-Institute for Molecular Medicine Finland, Helsinki, Finland

244 THL-National Institute for Health and Welfare, Helsinki, Finland

245 Iwate Tohoku Medical Megabank Organization, Iwate Medical University, Iwate, Japan

246 BHF Glasgow Cardiovascular Research Centre, Faculty of Medicine, Glasgow, UK

247 deCODE Genetics/Amgen, Inc., Reykjavik, Iceland

248 Icelandic Heart Association, Reykjavik, Iceland

249 Institute of Biomedicine, the Sahlgrenska Academy at University of Gothenburg, Goteborg, Sweden

250 Department of Epidemiology, University of Maryland School of Medicine, Baltimore, MD, USA

251 Institute of Cardiovascular and Medical Sciences, Faculty of Medicine, University of Glasgow, Glasgow, UK

252 Chair of Genetic Epidemiology, IBE, Faculty of Medicine, LMU Munich, Germany

253 Division of Epidemiology and Prevention, Aichi Cancer Center Research Institute, Nagoya, Japan

254 Department of Epidemiology, Nagoya University Graduate School of Medicine, Nagoya, Japan

255 University Medicine Greifswald, Institute for Community Medicine, SHIP-KEF, Greifswald, Germany

256 Department of Neurology, Caen University Hospital, Caen, France

257 University of Caen Normandy, Caen, France

258 Department of Internal Medicine, Erasmus University Medical Center, Rotterdam, Netherlands

259 Landspitali University Hospital, Reykjavik, Iceland

260 Survey Research Center, University of Michigan, Ann Arbor, MI, USA

261 University of Virginia Department of Neurology, Charlottesville, VA, USA

**References**

Barban, N., Jansen, R., De Vlaming, R., Vaez, A., Mandemakers, J.J., Tropf, F.C., Shen, X., Wilson, J.F., Chasman, D.I., Nolte, I.M., Tragante, V., Van Der Laan, S.W., Perry, J.R.B., Kong, A., Ahluwalia, T.S., Albrecht, E., Yerges-Armstrong, L., Atzmon, G., Auro, K., Ayers, K., Bakshi, A., Ben-Avraham, D., Berger, K., Bergman, A., Bertram, L., Bielak, L.F., Bjornsdottir, G., Bonder, M.J., Broer, L., Bui, M., Barbieri, C., Cavadino, A., Chavarro, J.E., Turman, C., Concas, M.P., Cordell, H.J., Davies, G., Eibich, P., Eriksson, N., Esko, T., Eriksson, J., Falahi, F., Felix, J.F., Fontana, M.A., Franke, L., Gandin, I., Gaskins, A.J., Gieger, C., Gunderson, E.P., Guo, X.Q., Hayward, C., He, C.Y., Hofer, E., Huang, H.Y., Joshi, P.K., Kanoni, S., Karlsson, R., Kiechl, S., Kifley, A., Kluttig, A., Kraft, P., Lagou, V., Lecoeur, C., Lahti, J., Li-Gao, R.F., Lind, P.A., Liu, T., Makalic, E., Mamasoula, C., Matteson, L., Mbarek, H., Mcardle, P.F., Mcmahon, G., Meddens, S.F.W., Mihailov, E., Miller, M., Missmer, S.A., Monnereau, C., Van Der Most, P.J., Myhre, R., Nalls, M.A., Nutile, T., Kalafati, I.P., Porcu, E., Prokopenko, I., Rajan, K.B., Rich-Edwards, J., Rietveld, C.A., Robino, A., Rose, L.M., Rueedi, R., Ryan, K.A., Saba, Y., Schmidt, D., Smith, J.A., Stolk, L., Streeten, E., Tonjes, A., Thorleifsson, G., Ulivi, S., et al. (2016). Genome-wide analysis identifies 12 loci influencing human reproductive behavior. *Nature Genetics* 48**,** 1462-1472.

Berndt, S.I., Gustafsson, S., Magi, R., Ganna, A., Wheeler, E., Feitosa, M.F., Justice, A.E., Monda, K.L., Croteau-Chonka, D.C., Day, F.R., Esko, T., Fall, T., Ferreira, T., Gentilini, D., Jackson, A.U., Luan, J.A., Randall, J.C., Vedantam, S., Willer, C.J., Winkler, T.W., Wood, A.R., Workalemahu, T., Hu, Y.-J., Lee, S.H., Liang, L., Lin, D.-Y., Min, J.L., Neale, B.M., Thorleifsson, G., Yang, J., Albrecht, E., Amin, N., Bragg-Gresham, J.L., Cadby, G., Den Heijer, M., Eklund, N., Fischer, K., Goel, A., Hottenga, J.-J., Huffman, J.E., Jarick, I., Johansson, A., Johnson, T., Kanoni, S., Kleber, M.E., Konig, I.R., Kristiansson, K., Kutalik, Z., Lamina, C., Lecoeur, C., Li, G., Mangino, M., Mcardle, W.L., Medina-Gomez, C., Muller-Nurasyid, M., Ngwa, J.S., Nolte, I.M., Paternoster, L., Pechlivanis, S., Perola, M., Peters, M.J., Preuss, M., Rose, L.M., Shi, J., Shungin, D., Smith, A.V., Strawbridge, R.J., Surakka, I., Teumer, A., Trip, M.D., Tyrer, J., Van Vliet-Ostaptchouk, J.V., Vandenput, L., Waite, L.L., Zhao, J.H., Absher, D., Asselbergs, F.W., Atalay, M., Attwood, A.P., Balmforth, A.J., Basart, H., Beilby, J., Bonnycastle, L.L., Brambilla, P., Bruinenberg, M., Campbell, H., Chasman, D.I., Chines, P.S., Collins, F.S., Connell, J.M., Cookson, W.O., De Faire, U., De Vegt, F., Dei, M., Dimitriou, M., Edkins, S., Estrada, K., Evans, D.M., Farrall, M., Ferrario, M.M., et al. (2013). Genome-wide meta-analysis identifies 11 new loci for anthropometric traits and provides insights into genetic architecture. *Nature Genetics* 45**,** 501-512.

Bradfield, J.P., Vogelezang, S., Felix, J.F., Chesi, A., Helgeland, , Horikoshi, M., Karhunen, V., Lowry, E., Cousminer, D.L., Ahluwalia, T.S., Thiering, E., Boh, E.T.-H., Zafarmand, M.H., Vilor-Tejedor, N., Wang, C.A., Joro, R., Chen, Z., Gauderman, W.J., Pitkänen, N., Parra, E.J., Fernandez-Rhodes, L., Alyass, A., Monnereau, C., Curtin, J.A., Have, C.T., Mccormack, S.E., Hollensted, M., Frithioff-Bøjsøe, C., Valladares-Salgado, A., Peralta-Romero, J., Teo, Y.-Y., Standl, M., Leinonen, J.T., Holm, J.-C., Peters, T., Vioque, J., Vrijheid, M., Simpson, A., Custovic, A., Vaudel, M., Canouil, M., Lindi, V., Atalay, M., Kähönen, M., Raitakari, O.T., Van Schaik, B.D.C., Berkowitz, R.I., Cole, S.A., Voruganti, V.S., Wang, Y., Highland, H.M., Comuzzie, A.G., Butte, N.F., Justice, A.E., Gahagan, S., Blanco, E., Lehtimäki, T., Lakka, T.A., Hebebrand, J., Bonnefond, A., Grarup, N., Froguel, P., Lyytikäinen, L.-P., Cruz, M., Kobes, S., Hanson, R.L., Zemel, B.S., Hinney, A., Teo, K.K., Meyre, D., North, K.E., Gilliland, F.D., Bisgaard, H., Bustamante, M., Bonnelykke, K., Pennell, C.E., Rivadeneira, F., Uitterlinden, A.G., Baier, L.J., Vrijkotte, T.G.M., Heinrich, J., Sørensen, T.I.A., Saw, S.-M., Pedersen, O., Hansen, T., Eriksson, J., Widén, E., Mccarthy, M.I., Njølstad, P.R., Power, C., Hyppönen, E., Sebert, S., Brown, C.D., Järvelin, M.-R., Timpson, N.J., Johansson, S., Hakonarson, H., Jaddoe, V.W.V., and Consortium, E.G.G. (2019). A trans-ancestral meta-analysis of genome-wide association studies reveals loci associated with childhood obesity. *Human Molecular Genetics*.

Brion, M.-J.A., Shakhbazov, K., and Visscher, P.M. (2013). Calculating statistical power in Mendelian randomization studies. *International Journal of Epidemiology* 42**,** 1497-1501.

Brockwell, S.E., and Gordon, I.R. (2001). A comparison of statistical methods for meta-analysis. *Statistics in Medicine* 20**,** 825-840.

Bulik-Sullivan, B., Finucane, H.K., Anttila, V., Gusev, A., Day, F.R., Loh, P.-R., Reprogen, C., Psychiatric Genomics, C., Genetic Consortium for Anorexia Nervosa of the Wellcome Trust Case Control, C., Duncan, L., Perry, J.R.B., Patterson, N., Robinson, E.B., Daly, M.J., Price, A.L., and Neale, B.M. (2015). An atlas of genetic correlations across human diseases and traits. *Nature Genetics* 47**,** 1236-1241.

Burgess, S., Small, D.S., and Thompson, S.G. (2017). A review of instrumental variable estimators for Mendelian randomization. *Statistical Methods in Medical Research* 26**,** 2333-2355.

Burgess, S., and Thompson, S.G. (2015). Multivariable Mendelian Randomization: The Use of Pleiotropic Genetic Variants to Estimate Causal Effects. *American Journal of Epidemiology* 181**,** 251-260.

Burgess, S., and Thompson, S.G. (2017). Interpreting findings from Mendelian randomization using the MR-Egger method. *European Journal of Epidemiology* 32**,** 377-389.

Bycroft, C., Freeman, C., Petkova, D., Band, G., Elliott, L.T., Sharp, K., Motyer, A., Vukcevic, D., Delaneau, O., O’connell, J., Cortes, A., Welsh, S., Young, A., Effingham, M., Mcvean, G., Leslie, S., Allen, N., Donnelly, P., and Marchini, J. (2018). The UK Biobank resource with deep phenotyping and genomic data. *Nature* 562**,** 203-209.

Cousminer, D.L., Berry, D.J., Timpson, N.J., Ang, W., Thiering, E., Byrne, E.M., Taal, H.R., Huikari, V., Bradfield, J.P., Kerkhof, M., Groen-Blokhuis, M.M., Kreiner-Møller, E., Marinelli, M., Holst, C., Leinonen, J.T., Perry, J.R.B., Surakka, I., Pietiläinen, O., Kettunen, J., Anttila, V., Kaakinen, M., Sovio, U., Pouta, A., Das, S., Lagou, V., Power, C., Prokopenko, I., Evans, D.M., Kemp, J.P., St Pourcain, B., Ring, S., Palotie, A., Kajantie, E., Osmond, C., Lehtimäki, T., Viikari, J.S., Kähönen, M., Warrington, N.M., Lye, S.J., Palmer, L.J., Tiesler, C.M.T., Flexeder, C., Montgomery, G.W., Medland, S.E., Hofman, A., Hakonarson, H., Guxens, M., Bartels, M., Salomaa, V., Consortium, T.R., Murabito, J.M., Kaprio, J., Sørensen, T.I.A., Ballester, F., Bisgaard, H., Boomsma, D.I., Koppelman, G.H., Grant, S.F.A., Jaddoe, V.W.V., Martin, N.G., Heinrich, J., Pennell, C.E., Raitakari, O.T., Eriksson, J.G., Smith, G.D., Hyppönen, E., Järvelin, M.-R., Mccarthy, M.I., Ripatti, S., Widén, E., and Consortium, F.T.E.G.G. (2013). Genome-wide association and longitudinal analyses reveal genetic loci linking pubertal height growth, pubertal timing and childhood adiposity. *Human Molecular Genetics* 22**,** 2735-2747.

Cousminer, D.L., Stergiakouli, E., Berry, D.J., Ang, W., Groen-Blokhuis, M.M., Körner, A., Siitonen, N., Ntalla, I., Marinelli, M., Perry, J.R.B., Kettunen, J., Jansen, R., Surakka, I., Timpson, N.J., Ring, S., Mcmahon, G., Power, C., Wang, C., Kähönen, M., Viikari, J., Lehtimäki, T., Middeldorp, C.M., Hulshoff Pol, H.E., Neef, M., Weise, S., Pahkala, K., Niinikoski, H., Zeggini, E., Panoutsopoulou, K., Bustamante, M., Penninx, B.W.J.H., Murabito, J., Torrent, M., Dedoussis, G.V., Kiess, W., Boomsma, D.I., Pennell, C.E., Raitakari, O.T., Hyppönen, E., Davey Smith, G., Ripatti, S., Mccarthy, M.I., and Widén, E. (2014). Genome-wide association study of sexual maturation in males and females highlights a role for body mass and menarche loci in male puberty. *Human Molecular Genetics* 23**,** 4452-4464.

Dersimonian, R., and Laird, N. (1986). Meta-analysis in clinical trials. *Controlled Clinical Trials* 7**,** 177-188.

Dupuis, J., Langenberg, C., Prokopenko, I., Saxena, R., Soranzo, N., Jackson, A.U., Wheeler, E., Glazer, N.L., Bouatia-Naji, N., and Gloyn, A.L. (2010). New genetic loci implicated in fasting glucose homeostasis and their impact on type 2 diabetes risk. *Nature Genetics* 42**,** 105-116.

Eriksson, J.G., Forsen, T., Tuomilehto, J., Osmond, C., and Barker, D.J.P. (2000). Early Growth, Adult Income, and Risk of Stroke. *Stroke* 31**,** 869-874.

Felix, J.F., Bradfield, J.P., Monnereau, C., Van Der Valk, R.J.P., Stergiakouli, E., Chesi, A., Gaillard, R., Feenstra, B., Thiering, E., Kreiner-Møller, E., Mahajan, A., Pitkänen, N., Joro, R., Cavadino, A., Huikari, V., Franks, S., Groen-Blokhuis, M.M., Cousminer, D.L., Marsh, J.A., Lehtimäki, T., Curtin, J.A., Vioque, J., Ahluwalia, T.S., Myhre, R., Price, T.S., Vilor-Tejedor, N., Yengo, L., Grarup, N., Ntalla, I., Ang, W., Atalay, M., Bisgaard, H., Blakemore, A.I., Bonnefond, A., Carstensen, L., Bone Mineral Density in Childhood, S., Early, G., Lifecourse Epidemiology, C., Eriksson, J., Flexeder, C., Franke, L., Geller, F., Geserick, M., Hartikainen, A.-L., Haworth, C.M.A., Hirschhorn, J.N., Hofman, A., Holm, J.-C., Horikoshi, M., Hottenga, J.J., Huang, J., Kadarmideen, H.N., Kähönen, M., Kiess, W., Lakka, H.-M., Lakka, T.A., Lewin, A.M., Liang, L., Lyytikäinen, L.-P., Ma, B., Magnus, P., Mccormack, S.E., Mcmahon, G., Mentch, F.D., Middeldorp, C.M., Murray, C.S., Pahkala, K., Pers, T.H., Pfäffle, R., Postma, D.S., Power, C., Simpson, A., Sengpiel, V., Tiesler, C.M.T., Torrent, M., Uitterlinden, A.G., Van Meurs, J.B., Vinding, R., Waage, J., Wardle, J., Zeggini, E., Zemel, B.S., Dedoussis, G.V., Pedersen, O., Froguel, P., Sunyer, J., Plomin, R., Jacobsson, B., Hansen, T., Gonzalez, J.R., Custovic, A., Raitakari, O.T., Pennell, C.E., Widén, E., Boomsma, D.I., Koppelman, G.H., Sebert, S., Järvelin, M.-R., Hyppönen, E., Mccarthy, M.I., et al. (2016). Genome-wide association analysis identifies three new susceptibility loci for childhood body mass index. *Human Molecular Genetics* 25**,** 389-403.

Hartwig, F.P., Davey Smith, G., and Bowden, J. (2017). Robust inference in summary data Mendelian randomization via the zero modal pleiotropy assumption. *International Journal of Epidemiology* 46**,** 1985-1998.

Hill, W d., Hagenaars, Saskia p., Marioni, Riccardo e., Harris, Sarah e., Liewald, David c., Davies, G., Okbay, A., Mcintosh, Andrew m., Gale, Catharine r., and Deary, Ian j. (2016). Molecular Genetic Contributions to Social Deprivation and Household Income in UK Biobank. *Current Biology* 26**,** 3083-3089.

Hyppönen, E., Leon, D., Kenward, M., and Lithell, H. (2001). Prenatal growth and risk of occlusive and haemorrhagic stroke in Swedish men and women born 1915-29: historical cohort study. *Bmj* 323**,** 1033-1034.

Kaijser, M., Bonamy, A., Akre, O., Cnattingius, S., Granath, F., Norman, M., and Ekbom, A. (2008). Perinatal risk factors for ischemic heart disease. *Circulation* 117**,** 405-410.

Lawlor, D.A., Ronalds, G., Clark, H., Davey Smith, G., and Leon, D.A. (2005). Birth weight is inversely associated with incident coronary heart disease and stroke among individuals born in the 1950s: findings from the Aberdeen Children of the 1950s prospective cohort study. *Circulation* 112**,** 1414-1418.

Liu, M., Jiang, Y., Wedow, R., Li, Y., Brazel, D.M., Chen, F., Datta, G., Davila-Velderrain, J., Mcguire, D., Tian, C., Zhan, X., Agee, M., Alipanahi, B., Auton, A., Bell, R.K., Bryc, K., Elson, S.L., Fontanillas, P., Furlotte, N.A., Hinds, D.A., Hromatka, B.S., Huber, K.E., Kleinman, A., Litterman, N.K., Mcintyre, M.H., Mountain, J.L., Northover, C.a.M., Sathirapongsasuti, J.F., Sazonova, O.V., Shelton, J.F., Shringarpure, S., Tung, J.Y., Vacic, V., Wilson, C.H., Pitts, S.J., Mitchell, A., Skogholt, A.H., Winsvold, B.S., Sivertsen, B., Stordal, E., Morken, G., Kallestad, H., Heuch, I., Zwart, J.-A., Fjukstad, K.K., Pedersen, L.M., Gabrielsen, M.E., Johnsen, M.B., Skrove, M., Indredavik, M.S., Drange, O.K., Bjerkeset, O., Børte, S., Stensland, S., Choquet, H., Docherty, A.R., Faul, J.D., Foerster, J.R., Fritsche, L.G., Gordon, S.D., Haessler, J., Hottenga, J.-J., Huang, H., Jang, S.-K., Jansen, P.R., Ling, Y., Mägi, R., Matoba, N., Mcmahon, G., Mulas, A., Orrù, V., Palviainen, T., Pandit, A., Reginsson, G.W., Smith, J.A., Taylor, A.E., Turman, C., Willemsen, G., Young, H., Young, K.A., Zajac, G.J.M., Zhao, W., Zhou, W., Bjornsdottir, G., Boardman, J.D., Boehnke, M., Boomsma, D.I., Chen, C., Cucca, F., Davies, G.E., Eaton, C.B., Ehringer, M.A., Esko, T., Fiorillo, E., Gillespie, N.A., Gudbjartsson, D.F., Haller, T., Harris, K.M., Heath, A.C., Hewitt, J.K., et al. (2019a). Association studies of up to 1.2 million individuals yield new insights into the genetic etiology of tobacco and alcohol use. *Nature Genetics* 51**,** 237-244.

Liu, X., Helenius, D., Skotte, L., Beaumont, R.N., Wielscher, M., Geller, F., Juodakis, J., Mahajan, A., Bradfield, J.P., Lin, F.T.J., Vogelezang, S., Bustamante, M., Ahluwalia, T.S., Pitkänen, N., Wang, C.A., Bacelis, J., Borges, M.C., Zhang, G., Bedell, B.A., Rossi, R.M., Skogstrand, K., Peng, S., Thompson, W.K., Appadurai, V., Lawlor, D.A., Kalliala, I., Power, C., Mccarthy, M.I., Boyd, H.A., Marazita, M.L., Hakonarson, H., Hayes, M.G., Scholtens, D.M., Rivadeneira, F., Jaddoe, V.W.V., Vinding, R.K., Bisgaard, H., Knight, B.A., Pahkala, K., Raitakari, O., Helgeland, , Johansson, S., Njølstad, P.R., Fadista, J., Schork, A.J., Nudel, R., Miller, D.E., Chen, X., Weirauch, M.T., Mortensen, P.B., Børglum, A.D., Nordentoft, M., Mors, O., Hao, K., Ryckman, K.K., Hougaard, D.M., Kottyan, L.C., Pennell, C.E., Lyytikainen, L.-P., Bønnelykke, K., Vrijheid, M., Felix, J.F., Lowe, W.L., Grant, S.F.A., Hyppönen, E., Jacobsson, B., Jarvelin, M.-R., Muglia, L.J., Murray, J.C., Freathy, R.M., Werge, T.M., Melbye, M., Buil, A., and Feenstra, B. (2019b). Variants in the fetal genome near pro-inflammatory cytokine genes on 2q13 associate with gestational duration. *Nature Communications* 10**,** 3927.

Lu, Y.C., Day, F.R., Gustafsson, S., Buchkovich, M.L., Na, J.B., Bataille, V., Cousminer, D.L., Dastani, Z., Drong, A.W., Esko, T., Evans, D.M., Falchi, M., Feitosa, M.F., Ferreira, T., Hedman, A.K., Haring, R., Hysi, P.G., Iles, M.M., Justice, A.E., Kanoni, S., Lagou, V., Li, R., Li, X., Locke, A., Lu, C., Magi, R., Perry, J.R.B., Pers, T.H., Qi, Q.B., Sanna, M., Schmidt, E.M., Scott, W.R., Shungin, D., Teumer, A., Vinkhuyzen, A.a.E., Walker, R.W., Westra, H.J., Zhang, M.F., Zhang, W.H., Zhao, J.H., Zhu, Z.H., Afzal, U., Ahluwalia, T.S., Bakker, S.J.L., Bellis, C., Bonnefond, A., Borodulin, K., Buchman, A.S., Cederholm, T., Choh, A.C., Choi, H.J., Curran, J.E., De Groot, L., De Jager, P.L., Dhonukshe-Rutten, R.a.M., Enneman, A.W., Eury, E., Evans, D.S., Forsen, T., Friedrich, N., Fumeron, F., Garcia, M.E., Gartner, S., Han, B.G., Havulinna, A.S., Hayward, C., Hernandez, D., Hillege, H., Ittermann, T., Kent, J.W., Kolcic, I., Laatikainen, T., Lahti, J., Leach, I.M., Lee, C.G., Lee, J.Y., Liu, T., Liu, Y.F., Lobbens, S., Loh, M., Lyytikainen, L.P., Medina-Gomez, C., Michaelsson, K., Nalls, M.A., Nielson, C.M., Oozageer, L., Pascoe, L., Paternoster, L., Polasek, O., Ripatti, S., Sarzynski, M.A., Shin, C.S., Narancic, N.S., Spira, D., Srikanth, P., Steinhagen-Thiessen, E., Sung, Y.J., Swart, K.M.A., Taittonen, L., Tanaka, T., et al. (2016). New loci for body fat percentage reveal link between adiposity and cardiometabolic disease risk. *Nature Communications* 7**,** 10495.

Nikpay, M., Goel, A., Won, H.-H., Hall, L.M., Willenborg, C., Kanoni, S., Saleheen, D., Kyriakou, T., Nelson, C.P., Hopewell, J.C., Webb, T.R., Zeng, L., Dehghan, A., Alver, M., Armasu, S.M., Auro, K., Bjonnes, A., Chasman, D.I., Chen, S., Ford, I., Franceschini, N., Gieger, C., Grace, C., Gustafsson, S., Huang, J., Hwang, S.-J., Kim, Y.K., Kleber, M.E., Lau, K.W., Lu, X., Lu, Y., Lyytikäinen, L.-P., Mihailov, E., Morrison, A.C., Pervjakova, N., Qu, L., Rose, L.M., Salfati, E., Saxena, R., Scholz, M., Smith, A.V., Tikkanen, E., Uitterlinden, A., Yang, X., Zhang, W., Zhao, W., De Andrade, M., De Vries, P.S., Van Zuydam, N.R., Anand, S.S., Bertram, L., Beutner, F., Dedoussis, G., Frossard, P., Gauguier, D., Goodall, A.H., Gottesman, O., Haber, M., Han, B.-G., Huang, J., Jalilzadeh, S., Kessler, T., König, I.R., Lannfelt, L., Lieb, W., Lind, L., Lindgren, C.M., Lokki, M.-L., Magnusson, P.K., Mallick, N.H., Mehra, N., Meitinger, T., Memon, F.-U.-R., Morris, A.P., Nieminen, M.S., Pedersen, N.L., Peters, A., Rallidis, L.S., Rasheed, A., Samuel, M., Shah, S.H., Sinisalo, J., Stirrups, K.E., Trompet, S., Wang, L., Zaman, K.S., Ardissino, D., Boerwinkle, E., Borecki, I.B., Bottinger, E.P., Buring, J.E., Chambers, J.C., Collins, R., Cupples, L.A., Danesh, J., Demuth, I., Elosua, R., Epstein, S.E., Esko, T., Feitosa, M.F., et al. (2015). A comprehensive 1000 Genomes-based genome-wide association meta-analysis of coronary artery disease. *Nature Genetics* 47**,** 1121-1130.

berg, S., Cnattingius, S., Sandin, S., Lichtenstein, P., and Iliadou, A.N. (2011). Birth Weight Predicts Risk of Cardiovascular Disease Within Dizygotic but Not Monozygotic Twin Pairs: A Large Population-Based Co-Twin–Control Study. *Circulation* 123**,** 2792-2798.

Rich-Edwards, J.W., Kleinman, K., Michels, K.B., Stampfer, M.J., Manson, J.E., Rexrode, K.M., Hibert, E.N., and Willett, W.C. (2005). Longitudinal study of birth weight and adult body mass index in predicting risk of coronary heart disease and stroke in women. *Bmj* 330**,** 1115.

Richedwards, J.W., Stampfer, M.J., Manson, J.E., Rosner, B., Hankinson, S.E., Colditz, G.A., Willett, W.C., and Hennekens, C.H. (1997). Birth weight and risk of cardiovascular disease in a cohort of women followed up since 1976. *BMJ* 315**,** 396-400.

Rietveld, C.A., Esko, T., Davies, G., Pers, T.H., Turley, P., Benyamin, B., Chabris, C.F., Emilsson, V., Johnson, A.D., and Lee, J.J. (2014). Common genetic variants associated with cognitive performance identified using the proxy-phenotype method. *Proceedings of the National Academy of Sciences* 111**,** 13790-13794.

Rietveld, C.A., Medland, S.E., Derringer, J., Yang, J., Esko, T., Martin, N.W., Westra, H.-J., Shakhbazov, K., Abdellaoui, A., Agrawal, A., Albrecht, E., Alizadeh, B.Z., Amin, N., Barnard, J., Baumeister, S.E., Benke, K.S., Bielak, L.F., Boatman, J.A., Boyle, P.A., Davies, G., De Leeuw, C., Eklund, N., Evans, D.S., Ferhmann, R., Fischer, K., Gieger, C., Gjessing, H.K., Hägg, S., Harris, J.R., Hayward, C., Holzapfel, C., Ibrahim-Verbaas, C.A., Ingelsson, E., Jacobsson, B., Joshi, P.K., Jugessur, A., Kaakinen, M., Kanoni, S., Karjalainen, J., Kolcic, I., Kristiansson, K., Kutalik, Z., Lahti, J., Lee, S.H., Lin, P., Lind, P.A., Liu, Y., Lohman, K., Loitfelder, M., Mcmahon, G., Vidal, P.M., Meirelles, O., Milani, L., Myhre, R., Nuotio, M.-L., Oldmeadow, C.J., Petrovic, K.E., Peyrot, W.J., Polašek, O., Quaye, L., Reinmaa, E., Rice, J.P., Rizzi, T.S., Schmidt, H., Schmidt, R., Smith, A.V., Smith, J.A., Tanaka, T., Terracciano, A., Van Der Loos, M.J.H.M., Vitart, V., Völzke, H., Wellmann, J., Yu, L., Zhao, W., Allik, J., Attia, J.R., Bandinelli, S., Bastardot, F., Beauchamp, J., Bennett, D.A., Berger, K., Bierut, L.J., Boomsma, D.I., Bültmann, U., Campbell, H., Chabris, C.F., Cherkas, L., Chung, M.K., Cucca, F., De Andrade, M., De Jager, P.L., De Neve, J.-E., Deary, I.J., Dedoussis, G.V., Deloukas, P., Dimitriou, M., Eiríksdóttir, G., Elderson, M.F., Eriksson, J.G., et al. (2013). GWAS of 126,559 Individuals Identifies Genetic Variants Associated with Educational Attainment. *Science* 340**,** 1467-1471.

Risnes, K.R., Romundstad, P.R., Nilsen, T.I., Eskild, A., and Vatten, L.J. (2009). Placental weight relative to birth weight and long-term cardiovascular mortality: findings from a cohort of 31,307 men and women. *American journal of epidemiology* 170**,** 622-631.

Saxena, R., Hivert, M.-F., Langenberg, C., Tanaka, T., Pankow, J.S., Vollenweider, P., Lyssenko, V., Bouatia-Naji, N., Dupuis, J., and Jackson, A.U. (2010). Genetic variation in GIPR influences the glucose and insulin responses to an oral glucose challenge. *Nature Genetics* 42**,** 142-148.

Scott, R.A., Scott, L.J., Mägi, R., Marullo, L., Gaulton, K.J., Kaakinen, M., Pervjakova, N., Pers, T.H., Johnson, A.D., Eicher, J.D., Jackson, A.U., Ferreira, T., Lee, Y., Ma, C., Steinthorsdottir, V., Thorleifsson, G., Qi, L., Van Zuydam, N.R., Mahajan, A., Chen, H., Almgren, P., Voight, B.F., Grallert, H., Müller-Nurasyid, M., Ried, J.S., Rayner, W.N., Robertson, N., Karssen, L.C., Van Leeuwen, E.M., Willems, S.M., Fuchsberger, C., Kwan, P., Teslovich, T.M., Chanda, P., Li, M., Lu, Y., Dina, C., Thuillier, D., Yengo, L., Jiang, L., Sparso, T., Kestler, H.A., Chheda, H., Eisele, L., Gustafsson, S., Frånberg, M., Strawbridge, R.J., Benediktsson, R., Hreidarsson, A.B., Kong, A., Sigurðsson, G., Kerrison, N.D., Luan, J., An, Liang, L., Meitinger, T., Roden, M., Thorand, B., Esko, T., Mihailov, E., Fox, C., Liu, C.-T., Rybin, D., Isomaa, B., Lyssenko, V., Tuomi, T., Couper, D.J., Pankow, J.S., Grarup, N., Have, C.T., Jørgensen, M.E., Jørgensen, T., Linneberg, A., Cornelis, M.C., Van Dam, R.M., Hunter, D.J., Kraft, P., Sun, Q., Edkins, S., Owen, K.R., Perry, J.R.B., Wood, A.R., Zeggini, E., Tajes-Fernandes, J., Abecasis, G.R., Bonnycastle, L.L., Chines, P.S., Stringham, H.M., Koistinen, H.A., Kinnunen, L., Sennblad, B., Mühleisen, T.W., Nöthen, M.M., Pechlivanis, S., Baldassarre, D., Gertow, K., Humphries, S.E., Tremoli, E., Klopp, N., Meyer, J., et al. (2017). An Expanded Genome-Wide Association Study of Type 2 Diabetes in Europeans. *Diabetes* 66**,** 2888-2902.

Shungin, D., Winkler, T.W., Croteau-Chonka, D.C., Ferreira, T., Lockes, A.E., Maegi, R., Strawbridge, R.J., Pers, T.H., Fischer, K., Justice, A.E., Workalemahu, T., Wu, J.M.W., Buchkovich, M.L., Heard-Costa, N.L., Roman, T.S., Drong, A.W., Song, C., Gustafsson, S., Day, F.R., Esko, T., Fall, T., Kutalik, Z., Luan, J.A., Randall, J.C., Scherag, A., Vedantam, S., Wood, A.R., Chen, J., Fehrmann, R., Karjalainen, J., Kahali, B., Liu, C.-T., Schmidt, E.M., Absher, D., Amin, N., Anderson, D., Beekman, M., Bragg-Gresham, J.L., Buyske, S., Demirkan, A., Ehret, G.B., Feitosa, M.F., Goel, A., Jackson, A.U., Johnson, T., Kleber, M.E., Kristiansson, K., Mangino, M., Leach, I.M., Medina-Gomez, C., Palmer, C.D., Pasko, D., Pechlivaniss, S., Peters, M.J., Prokopenko, I., Stancakova, A., Sung, Y.J., Tanakam, T., Teumer, A., Van Vliet-Ostaptchouk, J.V., Yengo, L., Zhang, W., Albrecht, E., Arnlov, J., Arscott, G.M., Bandinelli, S., Barrett, A., Bellis, C., Bennett, A.J., Berne, C., Blueher, M., Buhringer, S., Bonnet, F., Boettcher, Y., Bruinenberg, M., Carba, D.B., Caspersen, I.H., Clarke, R., Daw, E.W., Deelen, J., Deelman, E., Delgado, G., Doney, A.S.F., Eklund, N., Erdos, M.R., Estrada, K., Eury, E., Friedrichs, N., Garcia, M.E., Giedraitis, V., Gigante, B., Go, A.S., Golay, A., Grallert, H., Grammer, T.B., Graessler, J., Grewal, J., Groves, C.J., Haller, T., Hallmans, G., et al. (2015). New genetic loci link adipose and insulin biology to body fat distribution. *Nature* 518**,** 187-196.

Smith, C., Ryckman, K., Barnabei, V.M., Howard, B., Isasi, C.R., Sarto, G., Tom, S.E., Van Horn, L., Wallace, R., and Robinson, J.G. (2016). The impact of birth weight on cardiovascular disease risk in the Women's Health Initiative. *Nutrition, Metabolism and Cardiovascular Diseases* 26**,** 239-245.

Taal, H.R., St Pourcain, B., Thiering, E., Das, S., Mook-Kanamori, D.O., Warrington, N.M., Kaakinen, M., Kreiner-Møller, E., Bradfield, J.P., Freathy, R.M., Geller, F., Guxens, M., Cousminer, D.L., Kerkhof, M., Timpson, N.J., Ikram, M.A., Beilin, L.J., Bønnelykke, K., Buxton, J.L., Charoen, P., Chawes, B.L.K., Eriksson, J., Evans, D.M., Hofman, A., Kemp, J.P., Kim, C.E., Klopp, N., Lahti, J., Lye, S.J., Mcmahon, G., Mentch, F.D., Müller-Nurasyid, M., O'reilly, P.F., Prokopenko, I., Rivadeneira, F., Steegers, E.a.P., Sunyer, J., Tiesler, C., Yaghootkar, H., The Cohorts For, H., Aging Research in Genetic Epidemiology, C., Breteler, M.M.B., Debette, S., Fornage, M., Gudnason, V., Launer, L.J., Van Der Lugt, A., Mosley Jr, T.H., Seshadri, S., Smith, A.V., Vernooij, M.W., Early, G., Lifecourse Epidemiology, C., Blakemore, A.I.F., Chiavacci, R.M., Feenstra, B., Fernandez-Banet, J., Grant, S.F.A., Hartikainen, A.-L., Van Der Heijden, A.J., Iñiguez, C., Lathrop, M., Mcardle, W.L., Mølgaard, A., Newnham, J.P., Palmer, L.J., Palotie, A., Pouta, A., Ring, S.M., Sovio, U., Standl, M., Uitterlinden, A.G., Wichmann, H.E., Vissing, N.H., Decarli, C., Van Duijn, C.M., Mccarthy, M.I., Koppelman, G.H., Estivill, X., Hattersley, A.T., Melbye, M., Bisgaard, H., Pennell, C.E., Widen, E., Hakonarson, H., Smith, G.D., Heinrich, J., Jarvelin, M.-R., Jaddoe, V.W.V., and Early Growth Genetics, C. (2012). Common variants at 12q15 and 12q24 are associated with infant head circumference. *Nature Genetics* 44**,** 532-538.

The 1000 Genomes Project Consortium (2015). A global reference for human genetic variation. *Nature* 526**,** 68-74.

Thompson, S.G., and Sharp, S.J. (1999). Explaining heterogeneity in meta-analysis: A comparison of methods. *Statistics in Medicine* 18**,** 2693-2708.

Van Der Valk, R.J.P., Kreiner-Møller, E., Kooijman, M.N., Guxens, M., Stergiakouli, E., Sääf, A., Bradfield, J.P., Geller, F., Hayes, M.G., Cousminer, D.L., Körner, A., Thiering, E., Curtin, J.A., Myhre, R., Huikari, V., Joro, R., Kerkhof, M., Warrington, N.M., Pitkänen, N., Ntalla, I., Horikoshi, M., Veijola, R., Freathy, R.M., Teo, Y.-Y., Barton, S.J., Evans, D.M., Kemp, J.P., St Pourcain, B., Ring, S.M., Davey Smith, G., Bergström, A., Kull, I., Hakonarson, H., Mentch, F.D., Bisgaard, H., Chawes, B., Stokholm, J., Waage, J., Eriksen, P., Sevelsted, A., Melbye, M., Van Duijn, C.M., Medina-Gomez, C., Hofman, A., De Jongste, J.C., Taal, H.R., Uitterlinden, A.G., Armstrong, L.L., Eriksson, J., Palotie, A., Bustamante, M., Estivill, X., Gonzalez, J.R., Llop, S., Kiess, W., Mahajan, A., Flexeder, C., Tiesler, C.M.T., Murray, C.S., Simpson, A., Magnus, P., Sengpiel, V., Hartikainen, A.-L., Keinanen-Kiukaanniemi, S., Lewin, A., Da Silva Couto Alves, A., Blakemore, A.I., Buxton, J.L., Kaakinen, M., Rodriguez, A., Sebert, S., Vaarasmaki, M., Lakka, T., Lindi, V., Gehring, U., Postma, D.S., Ang, W., Newnham, J.P., Lyytikäinen, L.-P., Pahkala, K., Raitakari, O.T., Panoutsopoulou, K., Zeggini, E., Boomsma, D.I., Groen-Blokhuis, M., Ilonen, J., Franke, L., Hirschhorn, J.N., Pers, T.H., Liang, L., Huang, J., Hocher, B., Knip, M., Saw, S.-M., Holloway, J.W., Melén, E., Grant, S.F.A., Feenstra, B., Lowe, W.L., Widén, E., et al. (2015). A novel common variant in DCST2 is associated with length in early life and height in adulthood. *Human Molecular Genetics* 24**,** 1155-1168.

Warrington, N.M., Richmond, R., Fenstra, B., Myhre, R., Gaillard, R., Paternoster, L., Wang, C.A., Beaumont, R.N., Das, S., and Murcia, M. (2018). Maternal and fetal genetic contribution to gestational weight gain. *International Journal of Obesity* 42**,** 775-784.

Wheeler, E., Leong, A., Liu, C.-T., Hivert, M.-F., Strawbridge, R.J., Podmore, C., Li, M., Yao, J., Sim, X., Hong, J., Chu, A.Y., Zhang, W., Wang, X., Chen, P., Maruthur, N.M., Porneala, B.C., Sharp, S.J., Jia, Y., Kabagambe, E.K., Chang, L.-C., Chen, W.-M., Elks, C.E., Evans, D.S., Fan, Q., Giulianini, F., Go, M.J., Hottenga, J.-J., Hu, Y., Jackson, A.U., Kanoni, S., Kim, Y.J., Kleber, M.E., Ladenvall, C., Lecoeur, C., Lim, S.-H., Lu, Y., Mahajan, A., Marzi, C., Nalls, M.A., Navarro, P., Nolte, I.M., Rose, L.M., Rybin, D.V., Sanna, S., Shi, Y., Stram, D.O., Takeuchi, F., Tan, S.P., Van Der Most, P.J., Van Vliet-Ostaptchouk, J.V., Wong, A., Yengo, L., Zhao, W., Goel, A., Martinez Larrad, M.T., Radke, D., Salo, P., Tanaka, T., Van Iperen, E.P.A., Abecasis, G., Afaq, S., Alizadeh, B.Z., Bertoni, A.G., Bonnefond, A., Böttcher, Y., Bottinger, E.P., Campbell, H., Carlson, O.D., Chen, C.-H., Cho, Y.S., Garvey, W.T., Gieger, C., Goodarzi, M.O., Grallert, H., Hamsten, A., Hartman, C.A., Herder, C., Hsiung, C.A., Huang, J., Igase, M., Isono, M., Katsuya, T., Khor, C.-C., Kiess, W., Kohara, K., Kovacs, P., Lee, J., Lee, W.-J., Lehne, B., Li, H., Liu, J., Lobbens, S., Luan, J.A., Lyssenko, V., Meitinger, T., Miki, T., Miljkovic, I., Moon, S., Mulas, A., Müller, G., et al. (2017). Impact of common genetic determinants of Hemoglobin A1c on type 2 diabetes risk and diagnosis in ancestrally diverse populations: A transethnic genome-wide meta-analysis. *PLoS Medicine* 14**,** e1002383.

Willer, C.J., Schmidt, E.M., Sengupta, S., Peloso, G.M., Gustafsson, S., Kanoni, S., Ganna, A., Chen, J., Buchkovich, M.L., Mora, S., Beckmann, J.S., Bragg-Gresham, J.L., Chang, H.Y., Demirkan, A., Den Hertog, H.M., Do, R., Donnelly, L.A., Ehret, G.B., Esko, T., Feitosa, M.F., Ferreira, T., Fischer, K., Fontanillas, P., Fraser, R.M., Freitag, D.F., Gurdasani, D., Heikkila, K., Hypponen, E., Isaacs, A., Jackson, A.U., Johansson, A., Johnson, T., Kaakinen, M., Kettunen, J., Kleber, M.E., Li, X.H., Luan, J.A., Lyytikainen, L.P., Magnusson, P.K.E., Mangino, M., Mihailov, E., Montasser, M.E., Muller-Nurasyid, M., Nolte, I.M., O'connell, J.R., Palmer, C.D., Perola, M., Petersen, A.K., Sanna, S., Saxena, R., Service, S.K., Shah, S., Shungin, D., Sidore, C., Song, C., Strawbridge, R.J., Surakka, I., Tanaka, T., Teslovich, T.M., Thorleifsson, G., Van Den Herik, E.G., Voight, B.F., Volcik, K.A., Waite, L.L., Wong, A., Wu, Y., Zhang, W.H., Absher, D., Asiki, G., Barroso, I., Been, L.F., Bolton, J.L., Bonnycastle, L.L., Brambilla, P., Burnett, M.S., Cesana, G., Dimitriou, M., Doney, A.S.F., Doring, A., Elliott, P., Epstein, S.E., Eyjolfsson, G.I., Gigante, B., Goodarzi, M.O., Grallert, H., Gravito, M.L., Groves, C.J., Hallmans, G., Hartikainen, A.L., Hayward, C., Hernandez, D., Hicks, A.A., Holm, H., Hung, Y.J., Illig, T., Jones, M.R., Kaleebu, P., Kastelein, J.J.P., Khaw, K.T., Kim, E., et al. (2013). Discovery and refinement of loci associated with lipid levels. *Nature Genetics* 45**,** 1274–1283.

Yang, J., Loos, R.J.F., Powell, J.E., Medland, S.E., Speliotes, E.K., Chasman, D.I., Rose, L.M., Thorleifsson, G., Steinthorsdottir, V., Magi, R., Waite, L., Vernon Smith, A., Yerges-Armstrong, L.M., Monda, K.L., Hadley, D., Mahajan, A., Li, G., Kapur, K., Vitart, V., Huffman, J.E., Wang, S.R., Palmer, C., Esko, T., Fischer, K., Hua Zhao, J., Demirkan, A., Isaacs, A., Feitosa, M.F., Luan, J.A., Heard-Costa, N.L., White, C., Jackson, A.U., Preuss, M., Ziegler, A., Eriksson, J., Kutalik, Z., Frau, F., Nolte, I.M., Van Vliet-Ostaptchouk, J.V., Hottenga, J.-J., Jacobs, K.B., Verweij, N., Goel, A., Medina-Gomez, C., Estrada, K., Lynn Bragg-Gresham, J., Sanna, S., Sidore, C., Tyrer, J., Teumer, A., Prokopenko, I., Mangino, M., Lindgren, C.M., Assimes, T.L., Shuldiner, A.R., Hui, J., Beilby, J.P., Mcardle, W.L., Hall, P., Haritunians, T., Zgaga, L., Kolcic, I., Polasek, O., Zemunik, T., Oostra, B.A., Juhani Junttila, M., Gronberg, H., Schreiber, S., Peters, A., Hicks, A.A., Stephens, J., Foad, N.S., Laitinen, J., Pouta, A., Kaakinen, M., Willemsen, G., Vink, J.M., Wild, S.H., Navis, G., Asselbergs, F.W., Homuth, G., John, U., Iribarren, C., Harris, T., Launer, L., Gudnason, V., O/'Connell, J.R., Boerwinkle, E., Cadby, G., Palmer, L.J., James, A.L., Musk, A.W., Ingelsson, E., Psaty, B.M., Beckmann, J.S., Waeber, G., Vollenweider, P., Hayward, C., Wright, A.F., Rudan, I., et al. (2012). FTO genotype is associated with phenotypic variability of body mass index. *Nature* 490**,** 267-272.

Yavorska, O.O., and Burgess, S. (2017). MendelianRandomization: an R package for performing Mendelian randomization analyses using summarized data. *International Journal of Epidemiology***,** dyx034.

Yengo, L., Sidorenko, J., Kemper, K.E., Zheng, Z., Wood, A.R., Weedon, M.N., Frayling, T.M., Hirschhorn, J., Yang, J., Visscher, P.M., and The, G.C. (2018). Meta-analysis of genome-wide association studies for height and body mass index in ~700000 individuals of European ancestry. *Human Molecular Genetics* 27**,** 3641-3649.

Zeng, P., and Zhou, X. (2019a). Causal Association Between Birth Weight and Adult Diseases: Evidence From a Mendelian Randomization Analysis. *Frontiers in Genetics* 10.

Zeng, P., and Zhou, X. (2019b). Causal effects of blood lipids on amyotrophic lateral sclerosis: a Mendelian randomization study. *Human Molecular Genetics* 28**,** 688-697.
